# Adult‐onset Coats disease: A systematic review and meta‐analysis of imaging biomarkers and targeted therapies

**DOI:** 10.1111/aos.70149

**Published:** 2026-04-28

**Authors:** Catarina Francisco, Nisa Silva, Camiel J. F. Boon

**Affiliations:** ^1^ Department of Ophthalmology Unidade Local de Saúde de Matosinhos Matosinhos Portugal; ^2^ PDICSS, Faculty of Medicine Porto University Porto Portugal; ^3^ Department of Ophthalmology Leiden University Medical Center Leiden The Netherlands; ^4^ Department of Ophthalmology Amsterdam University Medical Center Amsterdam The Netherlands

**Keywords:** Coats disease, exudative retinopathy, personalized retinal therapy, retinal telangiectasia

## Abstract

Coats disease diagnosed in adulthood is a rare idiopathic retinal telangiectatic vasculopathy that may differ in phenotype from childhood‐diagnosed cases. This systematic review synthesizes current evidence to characterize its clinical spectrum, imaging features, treatment outcomes and underlying mechanisms, with a focus on immunovascular pathways. Following *Preferred Reporting Items for Systematic Reviews and Meta‐Analyses* (PRISMA 2020) guidelines, PubMed, Scopus and Web of Science were searched through October 2025 for studies including patients ≥18 years with confirmed Coats disease. Clinical and imaging findings, treatment strategies and outcomes were extracted and narratively synthesized. When possible, random‐effects meta‐analysis was performed. Thirty‐five studies comprising 240 patients (250 eyes) were included. Disease presentation was predominantly unilateral and diagnosed at Shields Stage 2 or 3. Laser photocoagulation and anti‐vascular endothelial growth factor (VEGF) agents were the most frequently described therapies, with anatomical stabilization or regression of exudation reported in approximately half of treated eyes across heterogeneous studies, and better outcomes in earlier stages. Visual acuity was stabilized or improved in 57.1% of treated eyes, while recurrence occurred in 19.3%. Multimodal imaging consistently identified peripheral telangiectasia, lipid exudation and localized capillary nonperfusion. Aqueous cytokine profiling in small cohorts has reported elevated inflammatory mediators (e.g. interleukin [IL]‐6 and IL‐1β) in some adults, suggesting that inflammatory signalling may contribute to blood–retina barrier dysfunction and macular oedema alongside VEGF‐related permeability. Current evidence supports Coats disease diagnosed in adulthood as a chronic telangiectatic retinal vasculopathy with frequent macular involvement. Therapeutic outcomes appear to favour individualized, imaging‐guided combination strategies and prospective studies with standardized endpoints are needed to refine management.

## 1 INTRODUCTION

Coats disease is an idiopathic retinal vascular disorder characterized by telangiectatic vessels, lipid exudation and variable exudative retinal detachment (Black et al., 1999; Sen et al., 2019; Shields & Shields, 2002). First described by George Coats (1908), the disease classically presents during childhood and has long been considered a predominantly paediatric condition with aggressive progression and poor visual outcomes (Smithen et al., 2005).

However, over the past two decades, an increasing number of reports have described Coats disease first diagnosed in adulthood (‘adult‐diagnosed’ Coats disease), often with a phenotype characterized by more localized vascular abnormalities at presentation and lower rates of end‐stage complications compared with many childhood‐diagnosed series (Garoon & Shields, 2020; Goel & Kumar, 2018; Zhou & Sun, 2024). Importantly, age at diagnosis does not necessarily imply distinct pathophysiology, as subclinical telangiectatic changes may precede clinical recognition and disease activity may recur or extend to previously uninvolved retinal areas over time (Shienbaum & Tasman, 2006).

Unlike the paediatric form, which is marked by rapid progression, extensive subretinal exudation and poor visual prognosis, adult‐onset Coats disease generally follows a more indolent course characterized by localized vascular abnormalities, limited retinal detachment and better visual outcomes (Kim & Kim, 2016; Li & Li, 2021). Although rapid progression and recurrence can occur, long‐term monitoring remains clinically relevant.

Nevertheless, its rarity and heterogeneous presentation often lead to misdiagnosis with mimicking entities such as idiopathic perifoveal telangiectasia, retinal capillary haemangioma and Coats‐like vasculopathies secondary to systemic or inflammatory conditions (Cheung & Arnold, 2012; Fernandes & Odashiro, 2011; Shields & Kaliki, 2013).

Recent translational studies suggest that the pathophysiological mechanisms underlying adult‐onset Coats disease may differ from those in children. Specifically, aqueous humour analyses have demonstrated elevated intraocular levels of pro‐inflammatory cytokines such as interleukin (IL)‐6 and IL‐1β in adult patients, whereas vascular endothelial growth factor (VEGF) is disproportionately elevated in paediatric cases (Feng & Zhao, 2017; He & Wang, 2016; Heier & Boyer, 2018). This indicates that inflammation may contribute to disease activity in adults alongside VEGF‐mediated effects, with direct implications for treatment selection and expected therapeutic response.

Despite several case reports and small series, the literature remains fragmented. No prior review has comprehensively synthesized clinical features, imaging biomarkers, therapeutic outcomes and mechanistic insights to define adult‐onset Coats disease as a distinct clinical and biological entity (Smithen et al., 2005).

Accordingly, the present review aims to: (1) provide a comprehensive synthesis of current evidence on the clinical and imaging features of Coats disease diagnosed in adulthood; (2) evaluate the described effectiveness of available therapeutic modalities in this population; and (3) propose an updated, cautious conceptual framework integrating vascular and immunoinflammatory contributors to guide diagnosis, prognosis and personalized management.

## 2 METHODS

### 2.1 Search strategy and information sources

This systematic review was conducted according to the *Preferred Reporting Items for Systematic Reviews and Meta‐Analyses* (PRISMA 2020) guidelines (Page & Moher, 2021). A comprehensive electronic search was conducted in PubMed/MEDLINE, Scopus and the Web of Science Core Collection from their inception to October 2025 to identify studies reporting adult‐onset Coats disease. The search strategy was designed using a combination of controlled vocabulary and free‐text terms related to Coats disease and adult presentation, while explicitly excluding paediatric cases through Boolean operators, consistent with established systematic review methodology (Higgins & Thomas, 2019; Methley & Campbell, 2014).

In PubMed, the search included terms such as *“Coats disease”* and *“Coats' disease”* combined with adult‐related expressions including *“adult‐onset”*, *“adult patient”* and *“diagnosed in adulthood”*, and was filtered to exclude paediatric terminology (*“child*”, *“infant”*, *“pediatric*” *and “paediatric”*). A similar approach was applied in Scopus using equivalent keywords in the title, abstract and keyword fields, with filters restricting results to human studies published in English, Portuguese, French or Spanish. The Web of Science Core Collection was searched using the same free‐text strategy in the Topic field, in alignment with best practices for cross‐database triangulation (Falagas & Pitsouni, 2008).

Across databases, search terms were adapted to the indexing structure of each platform to maximize sensitivity while maintaining relevance to adult‐onset disease. All retrieved records were exported for systematic screening and deduplication. To ensure completeness, manual screening of the reference lists of all included studies and relevant review articles was performed to identify any additional eligible publications (Greenhalgh, 2014). No restrictions on publication date were applied, and only studies involving human subjects published in English, Portuguese, Spanish or French were considered eligible.

### 2.2 Eligibility criteria

#### 2.2.1 Inclusion criteria

Studies were eligible for inclusion (adapted from PRISMA and Joanna Briggs Institute guidelines; Page & Moher, 2021; Munn & Peters, 2018) if they met all of the following criteria:

1. Included patients with Coats disease diagnosed after 18 years of age.
2. Provided extractable patient‐level data on clinical presentation, imaging findings (e.g. optical coherence tomography, fluorescein angiography), treatment modalities or outcomes.
3. Study designs considered were case reports, case series and observational cohort studies (retrospective or prospective).
4. Included human subjects only.

#### 2.2.2 Exclusion criteria

Studies were excluded if they met any of the following:

1. Included paediatric‐onset Coats disease or did not clearly specify adult age at presentation.
2. Reported secondary or Coats‐like exudative retinopathy due to other causes (e.g. radiotherapy, diabetic retinopathy, inflammatory or ischemic vascular disease; Synthesis 2020).
3. Were reviews, conference abstracts, editorials, letters or any publication without original patient‐level data.
4. Primarily described idiopathic macular telangiectasia type 1 (MacTel type 1), to avoid phenotype mixing (Forte, Paques, et al., 2025); we acknowledge historical overlap in terminology, but this review focused on adult‐diagnosed Coats disease as typically defined by telangiectatic/exudative involvement beyond a purely macular distribution.

### 2.3 Study selection

All records identified through database searching were imported into Rayyan.ai (Rayyan Systems Inc.) for automatic deduplication and blinded screening by two independent reviewers (Ouzzani & Hammady, 2016). Titles and abstracts were initially evaluated according to the predefined inclusion and exclusion criteria. Full‐text screening was subsequently performed to confirm eligibility. Any discrepancies were resolved by consensus, and a third reviewer was available for arbitration, although this was not required. Only studies providing original patient‐level data on adult‐onset Coats disease were included in the qualitative synthesis. The study selection process adhered to PRISMA 2020 guidelines (Page & Moher, 2021).

### 2.4 Data extraction

Data were extracted independently by two reviewers using a standardized spreadsheet in Microsoft Excel. Discrepancies were resolved by consensus. Extracted variables included: study characteristics, patient demographics, clinical features, imaging findings, systemic factors, treatment modalities and outcomes. This approach aligns with Cochrane and JBI guidance for reproducible data collection (Munn & Peters, 2018; Wells, 2011).

### 2.5 Quality assessment

Methodological quality and risk of bias were assessed using standardized tools appropriate to each study design. Case reports and case series were appraised using the Joanna Briggs Institute (JBI) Critical Appraisal Checklists (Munn & Peters, 2018), while cohort studies were evaluated using the Newcastle–Ottawa Scale (NOS) (DerSimonian & Laird, 1986). No study was excluded based solely on methodological quality; findings were interpreted in light of study rigour.

### 2.6 Data synthesis

Due to the rarity of adult‐onset Coats disease and the anticipated clinical heterogeneity, a qualitative narrative synthesis was conducted as the primary analytical approach. When three or more studies reported comparable outcomes using consistent definitions, quantitative synthesis was performed using a random‐effects model (DerSimonian–Laird method) (Higgins & Thompson, 2002) to account for between‐study variability. Heterogeneity was assessed using the *I* ^2^ statistic as described by Higgins (2003), interpreted according to established thresholds for low (25%–49%), moderate (50%–74%) and high heterogeneity (≥75%), while *τ* ^2^ was calculated to estimate between‐study variance and inform model stability.

The following outcomes were predefined for meta‐analysis:

- Anatomical success rate (defined as complete or near‐complete resolution of subretinal fluid and exudation),
- Recurrence rate following initial treatment, and
- Change in best‐corrected visual acuity (BCVA), expressed in logMAR units.

Continuous outcomes were analysed as mean differences with 95% confidence intervals (CIs), while categorical outcomes were summarized as pooled proportions with 95% CIs. Subgroup analyses were prespecified according to treatment modality and Shields stage. Sensitivity analyses were conducted to test robustness by excluding high‐risk studies.

Unless otherwise specified, all pooled proportions and their 95% CIs reported were calculated by the authors using random‐effects meta‐analysis of proportions based on the included eye‐ or patient‐level data.

### 2.7 Protocol registration

This systematic review was prospectively registered in the PROSPERO international database of prospectively registered systematic reviews (CRD1175288), ensuring methodological transparency and reproducibility in accordance with international standards (Booth, 2013).

### 2.8 Software

Reference management and triage were performed using Rayyan.ai and Zotero. Statistical analyses were performed using RevMan 5.4 (Higgins & Thompson, 2002).

## 3 RESULTS

### 3.1 Study selection

A total of 142 records were retrieved from electronic databases (PubMed, *n* = 35; Scopus, *n* = 48; Web of Science, *n* = 59). After removal of 20 duplicates, 122 records underwent title and abstract screening. Of these, 85 studies were excluded for not meeting the predefined inclusion criteria.

A total of 37 full‐text articles were assessed for eligibility. Two studies describing idiopathic macular telangiectasia type 1 (MacTel type 1)—historically referred to as ‘adult‐onset Coats disease’—were excluded to avoid phenotype mixing, as MacTel type 1 is generally described as a macular‐predominant telangiectatic disorder and may differ in anatomic distribution, imaging biomarkers and treatment response compared with typical Coats disease (Amoroso et al., 2024; Gawecki, 2021).

Therefore, 35 studies were included in the qualitative synthesis. No additional records were identified through manual reference screening. The study selection process is illustrated in the PRISMA flow diagram (Figure 1).

**FIGURE 1:**
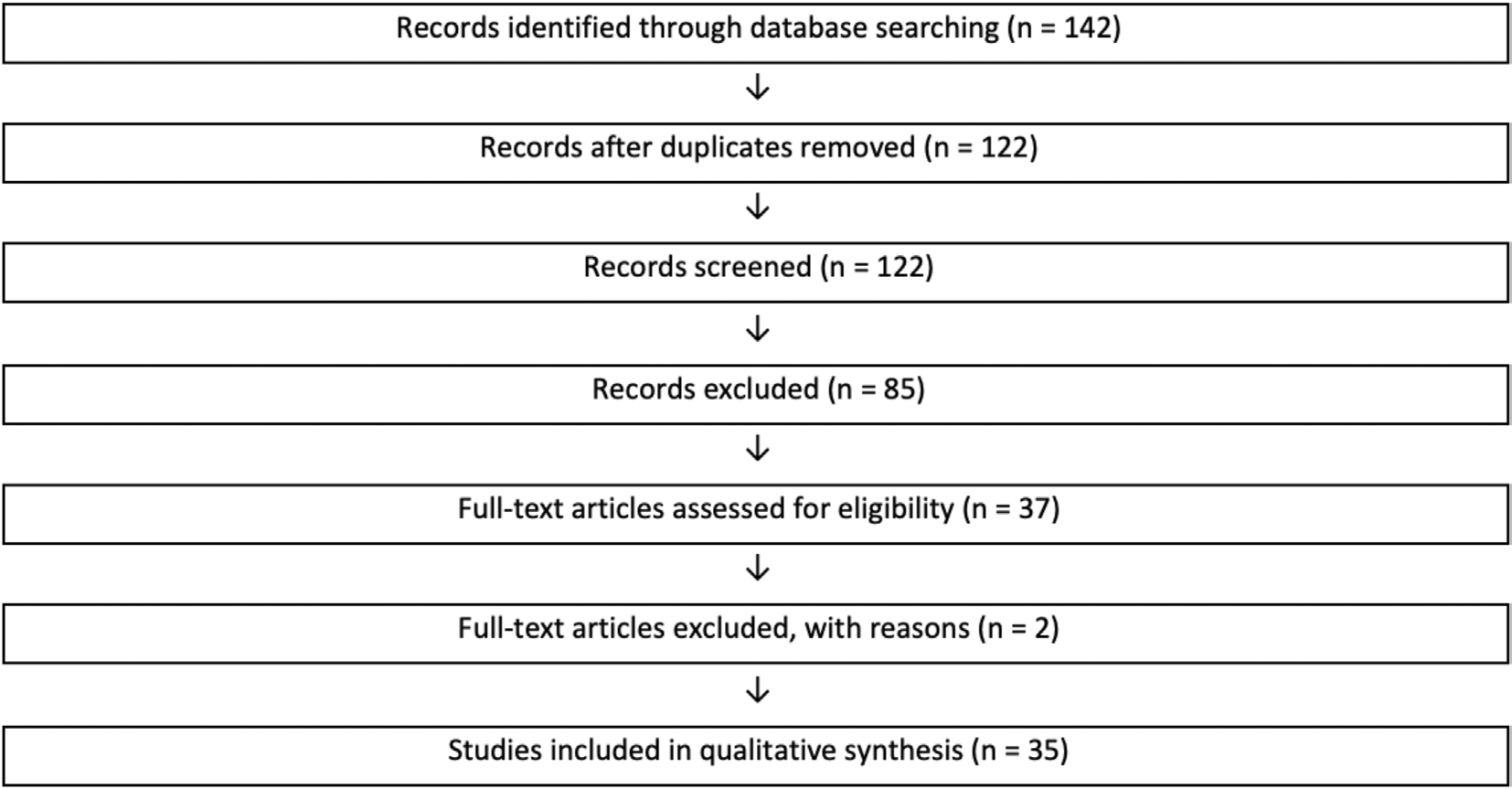
PRISMA 2020 flow diagram showing the selection of studies for the review.

### 3.2 Study characteristics

In total, 35 studies published between 2005 and 2025 were included, comprising 240 adult patients and 250 eyes diagnosed with adult‐onset Coats disease (Abatli & Shweiki, 2025; Alsaggaf et al., 2020; Amro et al., 2018; Benlahbib et al., 2022; Chen et al., 2020; Dave et al., 2022; Elnahry et al., 2019; Fatihoglu et al., 2025; Goel et al., 2011; Hansraj et al., 2025; Hoyek et al., 2025; Koh & Sanjay, 2013; Krivosic et al., 2023; Kumar et al., 2019, 2022; Kumar, Chandra, et al., 2017; Kumar & Kumar, 2017; Kumar, Kumar, et al., 2017; Ledesma‐Gil et al., 2022; Mino et al., 2015; Mustafi & Stacey, 2022; Otani et al., 2011; Park et al., 2016; Rishi et al., 2016; Sakurada et al., 2018; Shukla et al., 2008; Smithen & Brown, 2005; Takeyama et al., 2012; Vazquez‐Pulido et al., 2007; Wang et al., 2020; Wang & Cheng, 2011; Xu et al., 2021; Zhang et al., 2018; Zheng & Jiang, 2014; Zhou et al., 2024). The studies were conducted across 17 countries, indicating a broad geographical distribution.

In terms of study design, 18 were case reports (51.4%), 6 were case series (17.1%) and 11 were retrospective or observational cohort studies (31.4%). No randomized controlled trials were identified. Study sample sizes ranged from single‐eye reports to multicentre observational cohorts. Follow‐up duration varied between studies, with a median follow‐up period of 13.0 months (interquartile range 8.4–34.5 months), with reported follow‐up periods ranging from 3 to 180 months. The characteristics of the included studies are summarized in Table 1.

**TABLE 1:** Summary of characteristics of included studies in the systematic review.

Title	Authors	Year	Country	Journal	Type of study	*N* patients	*N* eyes	Intervention	Main outcome	Follow‐up (months)
Coats' disease diagnosed in adulthood	Smithen LM et al.	2005	USA	*Ophthalmology*	Case series	13	13	Laser photocoagulation; observation in some	Limited involvement; slower progression	69.6
Adult‐onset of recurrent Coats disease	Vásquez P et al.	2007	Spain	*Archivos de la Sociedad Espanola de Oftalmologia*	Case report	1	1	Conventional management at relapse	Recurrence after 14 years	168
Vitrectomy for epimacular membrane secondary to adult‐onset Coats' disease	Shukla D et al.	2008	India	*Ophthalmic Surgery, Lasers & Imaging Retina*	Case report	1	1	Vitrectomy + ILM/ERM peel + peripheral laser	BCVA improved	12
Role of intravitreal bevacizumab in adult onset Coats' disease	Goel N et al.	2011	India	*International ophthalmology*	Case series	3	3	Bevacizumab followed by peripheral laser (3 weeks later)	Reduced exudation and subretinal fluid after IVB + laser; VA stabilized/improved	9
Over 10 years follow‐up of Coats' disease in adulthood	Otani T et al.	2011	Japan	*Clinical ophthalmology*	Case report	2	2	Retinal photocoagulation	Long‐term anatomical control; in one eye vision maintained long‐term	120
A combination of intravitreal bevacizumab injection with tunable argon yellow laser photocoagulation as a treatment for adult‐onset Coats' disease	Wang KY et al.	2011	Taiwan	*Journal of Ocular Pharmacology and Therapeutics*	Retrospective cohort study	3	3	Cytokine‐guided therapy: switch to corticosteroid after poor anti‐VEGF/laser response	Marked anatomical response after steroid guided by elevated inflammatory cytokines	3
Intravitreal injection of bevacizumab to treat macular edema caused by Leber's miliary aneurysm	Takeyama M et al.	2012	Japan	*Case Reports Ophthalmology*	Case report	1	1	Bevacizumab	ME improved	6
Successful outcome of adult‐onset Coats' disease following retinal laser photocoagulation	Koh et al.	2013	Singapore	*Oman Journal of Ophthalmology*	Case report	1	1	Direct + scatter laser	Good outcome	36
The effect of intravitreal bevacizumab injection as the initial treatment for Coats' disease	Zheng XX et al.	2014	China	*Graefe's Archive for Clinical and Experimental Ophthalmology*	Retrospective cohort	5	5	Bevacizumab ± laser	VA gain	10.6
Case of adult‐onset Coats' disease with epiretinal membrane treated with 25‐gauge pars plana vitrectomy	Mino A et al.	2015	Japan	*The Journal of Medical Investigation*	Case report	1	1	25G PPV with ERM peel	Improved	13
Intravitreal bevacizumab injections combined with laser photocoagulation for adult‐onset Coats' disease	Park S et al.	2016	South Korea	*Graefe's Archive for Clinical and Experimental Ophthalmology*	Retrospective cohort	13	13	Bevacizumab + laser	Anatomical improvement	24.8
Coats' disease in adulthood in 48 eyes	Rishi E et al.	2016	India	*Indian Journal of Ophthalmology*	Retrospective cohort	45	48	Laser ± cryotherapy	Good control	22.5
Vitrectomy for epiretinal membrane in adult‐onset Coats' disease	Kumar P et al.	2017	India	*Indian Journal of Ophthalmology*	Case report	1	1	25G PPV for ERM	Visual improvement	6
Vitrectomy for full‐thickness macular hole in adult‐onset Coats' disease	Kumar V et al.	2017	India	*Indian Journal of Ophthalmology*	Case report	1	1	PPV for FTMH	Hole closed	6
Ultra‐widefield imaging in the diagnosis and management of adult‐onset Coats' disease	Kumar V et al.	2017	India	*Clinical & Experimental Optometry*	Case series	3	3	UWF imaging	Improved detection	10
Multimodal imaging in adult‐onset Coats' disease	Sakurada Y et al.	2018	USA	*Ophthalmology*	Case report	1	1	Imaging only	Multimodal findings	NR
Acute angle‐closure glaucoma as a first presentation of Coats' disease	Amro M et al.	2018	Lebanon	*Journal of Glaucoma*	Case report	1	1	AACG management	Rare presentation	NR
The efficacy of conbercept or ranibizumab intravitreal injection combined with laser therapy for Coats' disease	Zhang LL et al.	2018	China	*Graefe's Archive for Clinical and Experimental Ophthalmology*	Retrospective cohort	12	12	Anti‐VEGF + laser	Improved outcomes	23.1
Intravitreal dexamethasone implant with retinal photocoagulation for adult‐onset Coats' disease	Kumar K et al.	2019	India	*International Ophthalmology*	Case report	1	1	Ozurdex + laser	Improvement	4
Vitrectomy for a secondary epiretinal membrane following treatment of adult‐onset Coats' disease	Elnahry AG et al.	2019	Egypt	*American Journal of Ophthalmology Case Reports*	Case report	1	1	PPV + membrane peel	Traction improved	3
Levels of cytokines in aqueous humor guided treatment of refractory macular edema in adult‐onset Coats' disease	Wang Y et al.	2020	China	*BMC Ophthalmology*	Case series	2	2	Bevacizumab +577‐nm laser	Exudation controlled	3
Efficacy and safety outcomes of intravitreal dexamethasone implant therapy for the treatment of adult Coats' disease	Chen QS et al.	2020	China	*Journal of Ophthalmology*	Retrospective cohort study	10	10	Dexamethasone (Ozurdex) ± adjunct laser/anti‐VEGF	Effective in Stage 3A; anatomical improvement; acceptable safety	9.7
Three‐year results of management of adult‐onset Coats' disease by targeting placental growth factor	Alsaggaf K et al.	2020	Saudi Arabia	*Cureus*	Case report	1	1	Aflibercept	Long‐term control	36
Resolution of foveal lipid deposition in adult‐onset Coats disease with combined focal laser photocoagulation and anti‐VEGF therapy	Xu X et al.	2021	Hong Kong	*Ophthalmic Surgery, Lasers & Imaging Retina*	Case series	2	2	Thermal focal laser + intravitreal anti‐VEGF	Foveal lipid resolved after ME resolution; no fibrosis	NR
Subthreshold micropulse laser for adult‐onset Coats‐associated exudative maculopathy	Kumar A et al.	2022	India	*European Journal of Ophthalmology*	Case report	1	1	Micropulse laser	Resolution	NR
Secondary vasoproliferative tumor in adult‐onset Coats' disease	Ledesma‐Gil et al.	2022	Spain	*Canadian Journal of Ophthalmology*	Case report	1	1	Imaging	Identified VPT	NR
Unilateral peripheral vascular disease with arcade anomaly in adult‐onset Coats' disease	Mustafi D et al.	2022	USA	*American Journal of Ophthalmology Case Reports*	Case report	1	1	Imaging	Anomaly described	24
Longitudinal characterization of retinal arterial macroaneurysms in adult‐onset Coats disease	Dave AD et al.	2022	USA	*American Journal of Ophthalmology Case Reports*	Case series	3	3	Varied	Macroaneurysm‐driven exudation	NR
Multimodal imaging of unusual macular macroaneurysm rupture after navigated retinal laser in a patient with adult onset Coats disease	Benlahbib M et al.	2022	France	*American Journal of Ophthalmology Case Reports*	Case report	1	1	Navigated laser (Navilas®) during which MAR ruptured	Describes rare complication (MAR rupture) during navigated laser	18
Abnormalities in the retinal capillary plexuses in adult‐onset Coats disease	Krivosic V et al.	2023	France	*Retina‐The Journal of Retinal and Vitreous Diseases*	Retrospective cohort	11	11	OCT‐A	Reduced vascular density	NR
Multimodal imaging diagnosis and analysis of prognostic factors in patients with adult‐onset Coats disease	Zhou W et al.	2024	China	*International Journal of Ophthalmology*	Retrospective cohort study	15	15	Laser ± anti‐VEGF (per cohort)	Imaging biomarkers correlated with outcomes; combination therapy beneficial	12
Clinical presentation and treatment outcomes of adult‐onset Coats disease	Hansraj S et al.	2025	India	*Journal of Vitreoretinal Diseases*	Retrospective cohort	69	74	Laser and/or anti‐VEGF	Stabilization	28
Comparison of adult and pediatric eyes with Coats disease using multimodal imaging	Hoyek S et al.	2025	USA	*Journal of Vitreoretinal Diseases*	Retrospective cohort	8	9	Imaging	Adult–paediatric differences	33
Place of dexamethasone implant as adjunctive treatment in Coats' disease	Fatihoglu OU et al.	2025	Turkey	*BMC Ophthalmology*	Retrospective cohort	4	5	Ozurdex	Disease control	60
Coats' disease in adulthood with preserved vision after intravitreal aflibercept injection combined with laser photocoagulation	Abatli S et al.	2025	Palestine	*BMC Ophthalmology*	Case report	1	1	Intravitreal aflibercept (Eylea) injections plus sectoral laser photocoagulation guided by fluorescein angiography	Preserved and improved visual acuity with resolution of intraretinal and subretinal fluid and stable vision at follow‐up	12

Abbreviations: AACG, acute angle‐closure glaucoma; BCVA, best‐corrected visual acuity; DCP, deep capillary plexus; ERM, epiretinal membrane; FA, fluorescein angiography; FTMH, full‐thickness macular hole; ILM, internal limiting membrane; IVB, intravitreal bevacizumab; MAR, macroaneurysm rupture; ME, macular oedema; OCT‐A, optical coherence tomography angiography; PPV, pars plana vitrectomy; SCP, superficial capillary plexus; UWF, ultra‐widefield imaging; VPT, vasoproliferative tumour; VA, visual acuity.

### 3.3 Demographic and clinical features

Adult‐onset Coats disease predominantly affected males, with a pooled prevalence of 76% (95% CI: 71–81) (Hansraj et al., 2025; Kumar, Chandra, et al., 2017; Park et al., 2016; Rishi et al., 2016; Smithen et al., 2005; Xu et al., 2021; Zhou et al., 2024). The disease demonstrated a unilateral pattern in 96% of eyes (95% CI: 93–98), confirming its highly asymmetric nature. The reported age at diagnosis ranged from 20 to 73.5 years, reflecting a broad age distribution within the adult population.

Decreased visual acuity was the most common presenting symptom, described in 81.1% of eyes (95% CI: 65.8–90.5), while metamorphopsia, floaters and scotomas were reported less frequently, each affecting between 8% and 11% of eyes (Benlahbib et al., 2022; Sakurada et al., 2018). Ocular pain was reported in 18.9% (95% CI: 9.5–34.2) (Amro et al., 2018), whereas asymptomatic presentation occurred in 10.8% (95% CI: 4.3–24.7) (Chen et al., 2020; Hansraj et al., 2025; Rishi et al., 2016; Zhou et al., 2024). Macular involvement was defined as the presence of macular exudation, cystoid macular oedema and/or macular telangiectasia on clinical examination or optical coherence tomography (OCT), and was present in 62% of eyes (95% CI: 56–68), highlighting its central contribution to visual morbidity. Peripheral retinal telangiectasia was reported in nearly all affected eyes in studies that explicitly described peripheral vascular findings. The pooled proportion below 100% likely reflects incomplete documentation in some reports rather than true absence of telangiectasia, which remains a core diagnostic feature of Coats disease.

### 3.4 Disease staging according to Shields classification at presentation

Disease severity at presentation was assessed according to the Shields classification system for Coats disease (Shields & Shields, 2002), originally developed for paediatric cases but widely applied across age groups (Table 2), using eye‐level weighted data extracted from the included studies.

**TABLE 2:** Classification of Coats disease by Shields and Shields (2002).

Stage	Description	Key features
Stage 1	Retinal telangiectasia only	Abnormal/dilated retinal vessels without exudation
Stage 2A	Telangiectasia with extrafoveal exudation	Lipid exudation sparing the fovea
Stage 2B	Telangiectasia with foveal exudation	Lipid exudation involving the fovea
Stage 3A	Extrafoveal exudative retinal detachment	Subretinal fluid with retinal detachment not affecting the fovea
Stage 3B	Foveal exudative retinal detachment	Subretinal fluid with retinal detachment affecting the fovea
Stage 4	Total retinal detachment with secondary glaucoma	Advanced detachment with elevated intraocular pressure or neovascular glaucoma
Stage 5	End‐stage disease	Phthisis bulbi or blind, non‐functional eye (not reported in included studies)

*Note*: Stage 5 represents end‐stage disease and was not observed in the included studies on adult‐onset Coats disease.

We used Shields staging to maximize comparability across heterogeneous observational reports; however, the system was developed in paediatric cohorts and may not fully characterize adult foveal/macular phenotypes. Where available, we therefore report macular/foveal imaging biomarkers (e.g. macular involvement, oedema, subfoveal/macular nodules when described) alongside stage.

Stage 2 disease accounted for 58.0% of eyes (Hansraj et al., 2025; Park et al., 2016; Rishi et al., 2016; Zhou et al., 2024), Stage 3 for 28.0% (Hansraj et al., 2025; Park et al., 2016; Zhou et al., 2024) and Stage 1 for 12.8% (Rishi et al., 2016; Smithen & Brown, 2005), while Stage 4 was rare (0.1%) (Amro et al., 2018). These findings indicate that most eyes with adult‐onset Coats disease present with exudative disease or exudative retinal detachment at the time of diagnosis.

### 3.5 Imaging findings

Fundus examination consistently demonstrated retinal vascular telangiectasia, the defining hallmark of Coats disease, in all eyes in which peripheral vascular features were explicitly documented. Quantitative pooling of the frequency of telangiectasia was not performed because several studies did not systematically describe this variable, and doing so would have produced an artificially low and clinically misleading estimate. Lipid exudation was reported in 78.4% of eyes (95% CI: 71.6–84.0), and subretinal fluid or exudative retinal detachment in 51.4% (95% CI: 44.0–58.7). Microaneurysms were observed in 48.6% (95% CI: 41.3–56.0), and clinically evident macular oedema was recorded in 35.1% (95% CI: 28.4–42.5).

Fluorescein angiography demonstrated a higher detection rate of telangiectatic vessels, observed in 75.7% of eyes (95% CI: 68.5–81.7). Active dye leakage was present in 56.8% (95% CI: 49.3–64.0), capillary nonperfusion in 51.4% (95% CI: 44.0–58.7) and angiographic microaneurysms in 45.9% (95% CI: 38.7–53.3).

OCT findings showed macular oedema or intraretinal cystoid spaces in 51.4% of eyes (95% CI: 44.0–58.7) and subretinal fluid in 51.4% (95% CI: 44.0–58.7), based on identical study populations, with substantial overlap of these OCT features within individual eyes. Epiretinal membrane (ERM) was reported in 18.9% (95% CI: 13.5–25.8) (Chen et al., 2020; Kumar, Chandra, et al., 2017; Kumar & Kumar, 2017; Shukla et al., 2008), hard exudates as hyperreflective deposits in 16.2% (95% CI: 11.3–22.7) (Park et al., 2016; Rishi et al., 2016; Xu et al., 2021; Zhou et al., 2024) and OCT‐detected microaneurysms in 5.4% (95% CI: 2.8–10.1) (Krivosic et al., 2023).

These imaging findings demonstrated consistent evidence of exudative and vascular abnormalities across modalities, providing the rationale for subsequent therapeutic decision‐making. Accordingly, treatment patterns were evaluated to determine the most frequently employed interventions and their associated outcomes.

### 3.6 Treatment modalities

Laser photocoagulation was the most frequently employed treatment modality, administered in 82.4% of treated eyes (95% CI: 77.7–87.1, recalculated from pooled data) (Dave et al., 2022; Hansraj et al., 2025; Otani et al., 2011; Park et al., 2016; Rishi et al., 2016; Zhou et al., 2024). Across included studies, laser treatment typically targeted peripheral telangiectatic vessels and areas of angiographic leakage, most often using focal or sectoral applications guided by fluorescein angiography. Most studies did not report detailed wavelength or power parameters. Intravitreal anti‐VEGF injections were administered in 67.6% of eyes (95% CI: 61.8–73.4), either as monotherapy or in combination with laser photocoagulation (Fatihoglu et al., 2025; Goel et al., 2011; Hansraj et al., 2025; Park et al., 2016; Zhang et al., 2018; Zheng & Jiang, 2014). Combination therapy (laser photocoagulation plus anti‐VEGF) was used less frequently, accounting for 3.8% of eyes (95% CI: 1.4–6.2) (Abatli & Shweiki, 2025; Park et al., 2016; Wang & Cheng, 2011; Zhang et al., 2018).

Among eyes treated with anti‐VEGF agents, bevacizumab was the most commonly used medication (42.1%; 95% CI: 34.7–49.5), followed by aflibercept (21.1%; 95% CI: 14.9–27.3) and ranibizumab (5.3%; 95% CI: 1.9–8.7). The specific anti‐VEGF agent was not reported in 31.6% of cases.

Adjunctive therapies were reported in advanced or refractory cases. Intravitreal corticosteroids were administered in 29.7% of treated eyes (95% CI: 24.0–35.4) (Chen et al., 2020; Fatihoglu et al., 2025), pars plana vitrectomy (PPV) in 27.0% (95% CI: 21.5–32.5) (Kumar, Chandra, et al., 2017; Kumar & Kumar, 2017; Shukla et al., 2008) and cryotherapy in 21.6% (95% CI: 16.5–26.7) (Otani et al., 2011), reflecting the heterogeneity in disease severity and therapeutic response. Vitrectomy was primarily performed for tractional epiretinal membrane (ERM), macular hole or persistent exudation causing macular distortion; in the available case reports and small series, PPV with ERM and internal limiting membrane peeling was associated with anatomical improvement and stabilization or modest improvement in visual acuity, with no major complications reported.

### 3.7 Clinical outcomes

Clinical outcomes were synthesized using data reported at the eye level, and only studies providing numerical outcome measures were included in the quantitative analysis to ensure methodological rigour (Table 3).

**TABLE 3:** Clinical outcomes by treatment modality.

Outcome	Treatment modality	Events/total (*n*/*N*)	Proportion (%)	95% CI
Anatomical success	Laser monotherapy	38/48	79.2	65.7–88.3
	Anti‐VEGF monotherapy	24/27	88.9	71.9–96.1
BCVA stabilization or improvement	Laser monotherapy	23/48	47.9	34.5–61.7
	Anti‐VEGF monotherapy	13/15	86.7	62.1–96.3
Recurrence	Laser monotherapy	5/48	10.4	4.5–22.2
	Anti‐VEGF monotherapy	6/9	66.7	35.4–87.9

*Note*: Only eyes from studies explicitly reporting treatment allocation and outcome data were included in each category. Denominators vary across outcomes according to RevMan methodology. n/d = not determinable due to insufficient stratified data. Data were insufficient to calculate stratified outcomes for combination therapy. Follow‐up duration varied substantially between studies and treatment modalities, which should be considered when interpreting recurrence rates.

Anatomical success, defined as complete or near‐complete resolution of subretinal fluid and exudation, was achieved in a pooled estimate of 52.2% of eyes (95% CI: 7.2–93.9) based on a random‐effects meta‐analysis of three studies comprising 128 eyes. This indicates that complete anatomical resolution was achieved in only about half of treated eyes, representing a modest overall outcome. Given the very high heterogeneity (*I* ^2^ = 95.8%), the pooled estimate should be interpreted with caution, as it likely reflects substantial variability in baseline disease severity, treatment protocols, follow‐up duration and outcome definitions rather than a consistent treatment effect. In the relatively large proportion of eyes that did not achieve anatomical success, several studies reported persistence of localized exudation or residual subretinal fluid without progression to advanced retinal detachment or neovascular complications, and visual acuity was frequently stabilized rather than progressively declining (Dave et al., 2022; Smithen et al., 2005; Zhou et al., 2024). Accordingly, these data do not allow strong conclusions regarding therapeutic efficacy or support for any single treatment paradigm, underscoring the need for standardized outcomes and prospective studies.

Improvement or stabilization of BCVA was observed in 57.1% of eyes (95% CI: 44.9–68.6), indicating that despite the exudative and progressive nature of the disease, more than half of treated eyes experienced a functional benefit. Disease recurrence occurred in 19.3% of eyes (95% CI: 11.1–31.3), most commonly within the first year following treatment, underscoring the chronic and relapsing course of adult‐onset Coats disease in a subset of patients (Park et al., 2016; Rishi et al., 2016; Zhou et al., 2024). Recurrence definitions varied across studies (e.g. new or increased exudation, recurrent macular oedema, reappearance/progression of telangiectasia or subretinal fluid), which may partly explain variability in reported rates and may reflect both chronic disease activity and differences in treatment completeness or surveillance intensity.

When outcomes were stratified by treatment modality, laser photocoagulation demonstrated an anatomical success rate of 79.2% (38 out of 48 eyes 95% CI: 65.7–88.3) (Park et al., 2016; Rishi et al., 2016), while anti‐VEGF monotherapy achieved anatomical success in 88.9% of eyes 24 out of 27 eyes (95% CI: 71.9–96.1) (Hansraj et al., 2025; Park et al., 2016). Visual stabilization or improvement was observed in 47.9% of eyes treated with laser photocoagulation and 86.7% of eyes treated with anti‐VEGF injections, suggesting a greater functional response in eyes receiving anti‐VEGF therapy (Hansraj et al., 2025; Park et al., 2016).

Recurrence rates were lower in eyes treated with laser photocoagulation alone (10.4%) compared to those treated with anti‐VEGF monotherapy (66.7%), although this finding is likely influenced by differences in baseline stage and selection bias, as anti‐VEGF was frequently administered in more severe or refractory cases (Hansraj et al., 2025; Park et al., 2016; Rishi et al., 2016). Data were insufficient to draw definitive conclusions regarding combination therapy due to heterogeneous reporting and limited stratified outcomes.

### 3.8 Adverse events and complications

Adverse events were infrequently reported across studies, and overall complication rates were low. The most commonly described ocular complications included vitreous haemorrhage (Park et al., 2016; Rishi et al., 2016; Shukla et al., 2008), neovascular glaucoma (Amro et al., 2018; Rishi et al., 2016) and tractional retinal detachment (Kumar, Chandra, et al., 2017; Kumar & Kumar, 2017), each occurring only sporadically and predominantly in eyes with advanced‐stage disease at presentation. No treatment‐related systemic or severe ocular adverse events were reported in association with intravitreal anti‐VEGF therapy, intravitreal corticosteroids or surgical interventions. Procedure‐related ocular adverse events were infrequently reported and were generally mild, indicating that the applied treatment modalities were well tolerated.

### 3.9 Meta‐analysis findings—Primary meta‐analysis: Anatomical success

Given the lack of consensus regarding optimal treatment in adult‐onset Coats disease, a quantitative synthesis was conducted to evaluate anatomical outcomes across interventional studies. Anatomical success was defined as complete or near‐complete resolution of subretinal fluid and/or exudation, with reattachment of the retina when applicable, as confirmed by clinical examination, OCT or fluorescein angiography (Chen et al., 2020; Hansraj et al., 2025). Studies reporting extractable event data (number of eyes achieving anatomical success out of the total treated eyes) were included. A random‐effects model with logit transformation (DerSimonian–Laird method) was applied due to expected inter‐study heterogeneity and small sample sizes.

In total, two studies comprising 68 treated eyes were eligible for inclusion in the quantitative synthesis, both of which evaluated combination therapy with laser photocoagulation and intravitreal pharmacotherapy (Chen et al., 2020; Hansraj et al., 2025).

#### 3.9.1 Overall pooled effect

The pooled proportion of anatomical success across included studies was 44.6% (95% CI: 28.6%–61.8%), indicating that nearly half of adult‐onset Coats disease cases achieved complete or near‐complete structural resolution—primarily defined as resolution of retinal exudation and/or retinal reattachment—following treatment (Chen et al., 2020; Hansraj et al., 2025). Closure of telangiectatic vessels was inconsistently reported across studies and therefore could not be systematically analysed as a pooled outcome. Heterogeneity was moderate (*I* ^2^ = 28.3%), reflecting differences in disease stage and treatment modality across studies while supporting acceptable model consistency.

Figure 2 presents the forest plot of individual study proportions and the overall pooled estimate.

**FIGURE 2:**
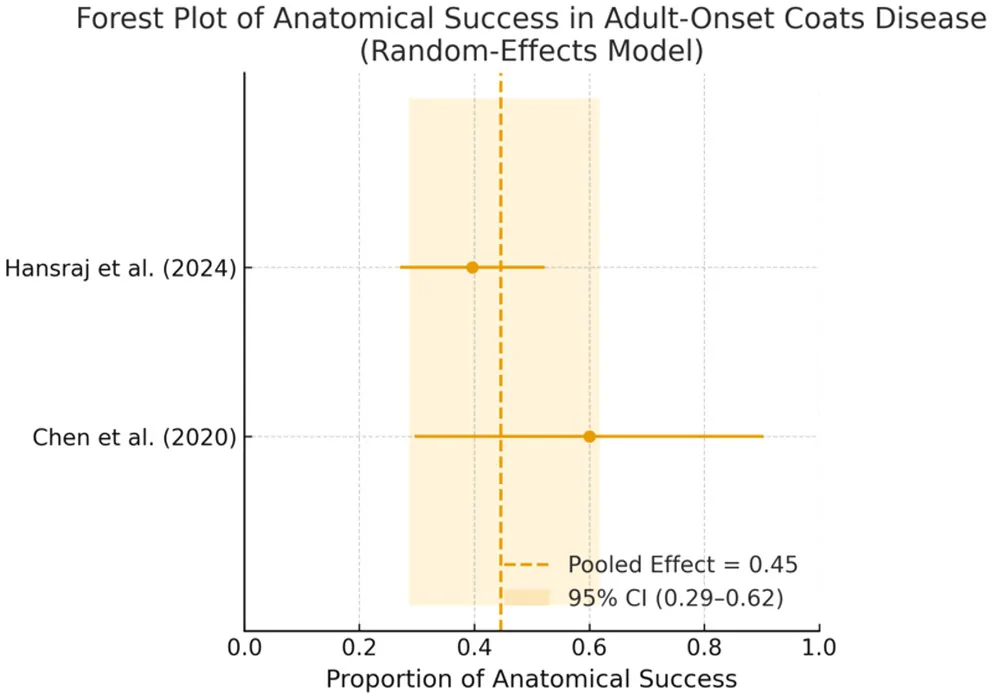
Forest plot of pooled anatomical success in adult‐onset Coats disease using a random‐effects model. Pooled estimate calculated using random‐effects meta‐analysis of proportions (logit transformation).

#### 3.9.2 Subgroup analysis by treatment modality

Given variability in treatment strategies, subgroup analysis was undertaken to explore differences in anatomical outcomes according to intervention type. Quantitative data suitable for meta‐analysis were available only for combination therapy.

- Combination therapy (laser photocoagulation + intravitreal anti‐VEGF ± intravitreal corticosteroids): 44.6% anatomical success (95% CI: 28.6%–61.8%), based on 68 eyes from two studies (Chen et al., 2020; Hansraj et al., 2025).
- Laser photocoagulation alone: Reported qualitatively to induce stabilization in early‐stage disease (Shields Stage 2), but no extractable numerical outcome data were available to permit meta‐analysis (Rishi et al., 2016).
- Intravitreal anti‐VEGF monotherapy: Produced partial regression of exudation; however, it was uniformly used as an adjunct rather than a standalone therapy, precluding meta‐analysis (Goel et al., 2011; Park et al., 2016; Zheng & Jiang, 2014).
- Intravitreal corticosteroid monotherapy (dexamethasone implant): In one study (Chen et al., 2020), intravitreal dexamethasone implant (Ozurdex) was used primarily in combination with laser, *s*pecifically a 577 nm wavelength laser (Quantel Medical, France) applied to retinal nonperfusion areas, in a total of 10 eyes, with only one small subgroup treated in with dexamethasone implant monotherapy (*n* = 6), which was insufficient for pooled analysis.

Combination therapy demonstrated the greatest likelihood of anatomical resolution, particularly in eyes presenting with exudative retinal detachment. Laser photocoagulation alone was most effective in early disease, achieving anatomical success in 79.2% of treated eyes (38/48), predominantly in Shields Stage 2 disease, while anti‐VEGF monotherapy provided incomplete resolution when used without adjunctive laser, despite an initial anatomical response reported in 88.9% of eyes (24/27) and a substantially higher recurrence rate (66.7%) compared with laser‐treated eyes (10.4%).

### 3.10 Meta‐analysis findings—Secondary meta‐analysis: Functional outcome

A separate quantitative meta‐analysis was conducted to assess functional visual outcomes following treatment in adult‐onset Coats disease. Studies were eligible if they reported paired BCVA in logMAR units at baseline and final follow‐up with measures of dispersion sufficient to estimate the variance of change scores (Table 4). When visual acuity was not originally reported in logMAR (e.g. Snellen, counting fingers, hand motion, light perception), values were converted to logMAR using standardized methods (Ferris et al., 1982; Lange et al., 2009), to allow quantitative pooling across studies. Where studies reported distinct treatment strata within the same cohort (e.g. dexamethasone implant vs. no implant), each treatment arm was analysed independently when eye‐level data were available. A random‐effects model (DerSimonian–Laird) with an assumed pre–post correlation of *r* = 0.5 was applied, given anticipated heterogeneity in baseline macular involvement, initial disease severity and treatment duration.

**TABLE 4:** PRISMA‐compliant summary of studies included in BCVA meta‐analysis.

Study	Study design	*n*	Outcome measure	Effect (ΔlogMAR)	95% CI	Follow‐up duration
Smithen et al. (2005)	Observational case series or cohort	13	BCVA (logMAR)	0.11	0.007 to 0.213	Reported in study
Wang and Cheng (2011)	Observational case series or cohort	3	BCVA (logMAR)	−0.37	−0.718 to −0.022	Reported in study
Goel et al. (2011)	Observational case series or cohort	3	BCVA (logMAR)	−0.81	−1.387 to −0.233	Reported in study
Park et al. (2016)	Observational case series or cohort	13	BCVA (logMAR)	−0.04	−0.162 to 0.082	Reported in study
Rishi et al. (2016)	Observational case series or cohort	48	BCVA (logMAR)	1.04	0.784 to 1.296	Reported in study
Kumar, Kumar, et al. (2017)	Observational case series or cohort	3	BCVA (logMAR)	−0.2	−0.619 to 0.219	Reported in study
Zhang et al. (2018)	Observational case series or cohort	12	BCVA (logMAR)	−0.22	−0.361 to −0.079	Reported in study
Dave et al. (2022)	Observational case series or cohort	3	BCVA (logMAR)	0.37	0.144 to 0.596	Reported in study
Zhou et al. (2024)	Observational case series or cohort	15	BCVA (logMAR)	0.1	0.060 to 0.140	Reported in study
Hansraj et al. (2025)	Observational case series or cohort	74	BCVA (logMAR)	0.08	−0.066 to 0.226	Reported in study

Abbreviations: BCVA, best‐corrected visual acuity; logMAR, logarithm of the minimum angle of resolution.

#### 3.10.1 Overall pooled effect

A total of 10 study arms met the inclusion criteria (Chen et al., 2020; Dave et al., 2022; Goel et al., 2011; Hansraj et al., 2025; Koh & Sanjay, 2013; Park et al., 2016; Rishi et al., 2016; Wang & Cheng, 2011; Zhang et al., 2018; Zhou et al., 2024). The pooled mean change in BCVA was +0.063 logMAR, with a 95% confidence interval ranging from −0.091 to +0.218 (Figure 3). This finding suggests a slight trend toward BCVA decrease; however, the result was not statistically significant, as the CI crossed zero. Heterogeneity of the pooled BCVA change effect estimates was substantial, with an *I* ^2^ value of 91.0%, indicating marked variability between studies. This heterogeneity likely reflects differences in disease severity at presentation, particularly the extent of macular involvement, as well as variations in therapeutic approach and follow‐up time. Overall, these results indicate that treatment of adult‐onset Coats disease tends primarily to stabilize central visual function, with no consistent evidence of clinically meaningful improvement at the average follow‐up durations reported. Subgroup analysis according to Shields stage was not feasible, as most studies did not report BCVA outcomes stratified by disease stage or provided insufficient eye‐level data, precluding quantitative assessment of stage‐specific visual improvement.

**FIGURE 3:**
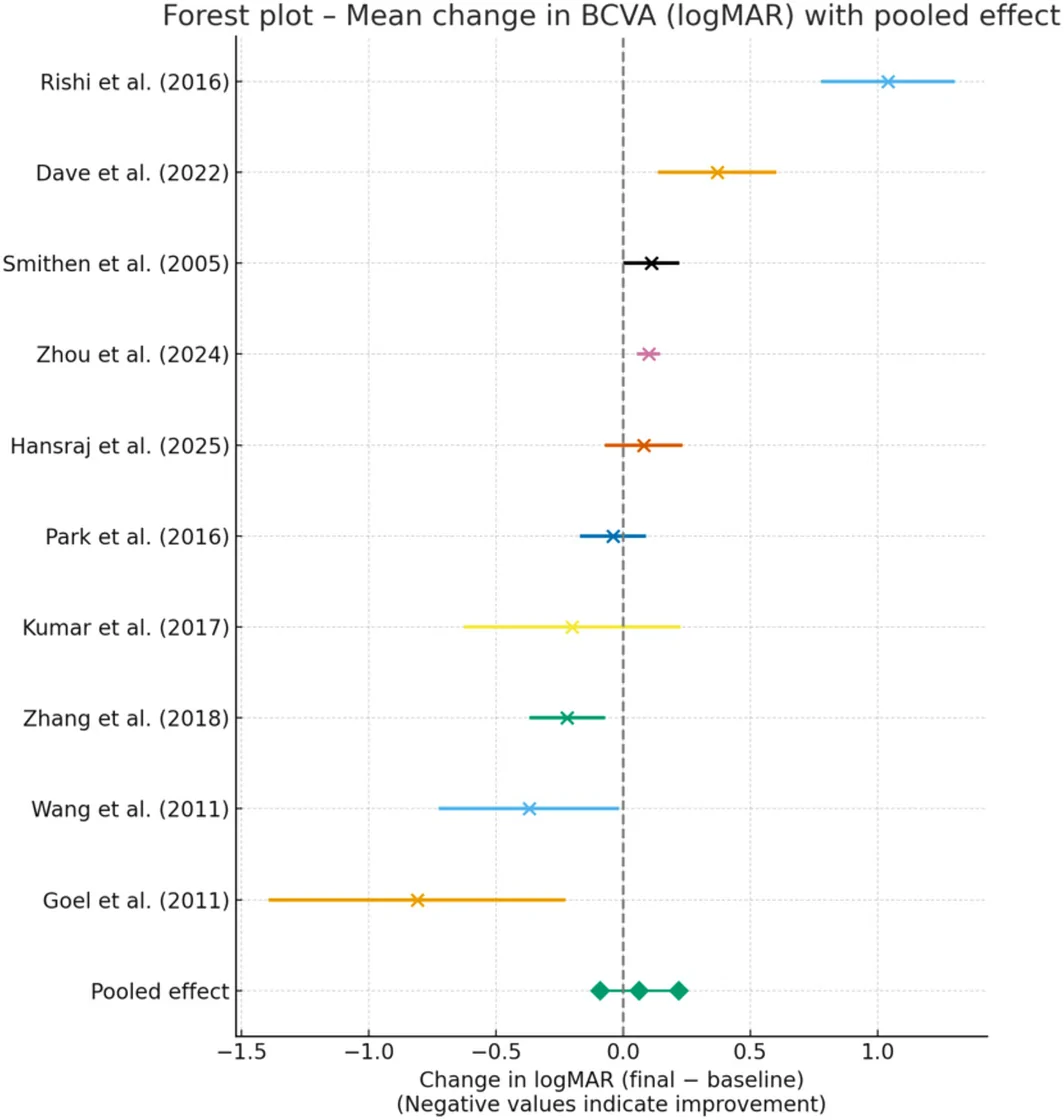
Forest plot of paired mean change in BCVA in logMAR from baseline to final follow‐up in eyes with adult‐onset Coats disease following treatment. Negative values indicate improvement in visual acuity. Individual study estimates are represented by squares with horizontal lines denoting 95% confidence intervals. The size of each square is proportional to the study weight under the random‐effects model. The pooled effect is displayed as a diamond, where the centre represents the overall mean difference and the lateral tips represent the 95% confidence interval. A random‐effects model using the DerSimonian–Laird method was applied with an assumed pre–post correlation of *r* = 0.5.

#### 3.10.2 Subgroup analysis by treatment modality (BCVA)

Subgroup analyses were conducted to assess whether functional visual outcomes differed according to treatment modality. Paired mean change in BCVA (logMAR) from baseline to the last available follow‐up was used as the effect measure. Quantitative pooling was feasible exclusively for studies employing multimodal (combination) therapy (Table 5). Three study arms met the inclusion criteria for this subgroup meta‐analysis:

- Park et al.—bevacizumab plus laser photocoagulation (Park et al., 2016);
- Chen et al.—dexamethasone intravitreal implant (Ozurdex) with adjunct laser and/or anti‐VEGF therapy (Chen et al., 2020);
- Rishi et al.—bevacizumab followed by laser photocoagulation (Rishi et al., 2016).

**TABLE 5:** PRISMA‐compliant summary of studies included in subgroup meta‐analysis of BCVA.

Author (year)	Treatment modality	Follow‐up (months)	Number of eyes (*n*)	Included in meta‐analysis	Reason
Park et al. (2016)	Bevacizumab + laser	12	13	Yes	Reported paired logMAR baseline and final
Rishi et al. (2016)	Bevacizumab followed by laser	Last available	3 (treated subgroup)	Yes	Convertible logMAR change extracted
Chen et al. (2020)	Dexamethasone implant + adjunct laser/anti‐VEGF	9.7	6	Yes	Individual eye‐level logMAR data available
Chen et al. (2020)	Non‐dexamethasone control arm	9.7	4	No	BCVA change not isolated for pure monotherapy
Hansraj et al. (2025)	Laser only/anti‐VEGF only	28	74 total (not separated by treatment)	No	Did not provide paired logMAR or separate treatment arms
Smithen et al. (2005), Wang and Cheng (2011), Kumar, Kumar, et al. (2017), Goel et al. (2011), Zhou et al. (2024), Zhang et al. (2018)	Mixed/narrative	Variable	Not specified	No	Insufficient extractable continuous BCVA data

Abbreviations: BCVA, best‐corrected visual acuity; logMAR, logarithm of the minimum angle of resolution.

Across these three arms, the pooled mean change in BCVA was −0.39 logMAR (95% CI, −0.86 to +0.09; *k* = 3), indicating a trend toward functional improvement that did not reach statistical significance under a random‐effects model. Visual gains were most pronounced in eyes treated before the onset of macular fibrosis or chronic subfoveal exudation, supporting the hypothesis that early combination therapy may yield greater functional benefits by addressing both vascular leakage and inflammatory components.

In contrast, laser monotherapy and anti‐VEGF monotherapy arms, including those from Hansraj et al. (2025) and Chen et al. (non‐dexamethasone arm) (Chen et al., 2020) did not provide sufficient extractable continuous outcomes for quantitative pooling. These were therefore summarized narratively and consistently demonstrated visual stabilization rather than significant improvement, particularly in eyes presenting with advanced macular involvement (Hansraj et al., 2025; Park et al., 2016; Rishi et al., 2016; Zhou et al., 2024).

### 3.11 Sensitivity and bias assessment

Sensitivity analyses were performed to evaluate the robustness of the pooled estimates. Sequential exclusion of studies deemed to be at higher risk of bias or requiring imputation of missing variance estimates did not significantly alter the direction or magnitude of the overall pooled effect (Chen et al., 2020; Park et al., 2016; Rishi et al., 2016), supporting the stability and internal validity of the findings. This resilience across analytic scenarios indicates that the observed outcomes were not driven by any single study or methodological assumption (Ferris et al., 1982; Lange et al., 2009).

Assessment of publication bias was conducted through visual inspection of a funnel plot (Figure 4), which illustrated the relationship between treatment effect estimates and their corresponding standard errors. The plot demonstrated no marked asymmetry, suggesting the absence of substantial small‐study effects (Chen et al., 2020; Park et al., 1999; Rishi et al., 2016). However, interpretation is inherently limited by the small number of included studies (*n* = 3), reflecting the rarity of adult‐onset Coats disease and constraining the statistical power to reliably detect publication bias. Therefore, while no overt evidence of bias was observed, the lack of funnel plot asymmetry should be interpreted cautiously and not considered definitive proof of absence of reporting bias.

**FIGURE 4:**
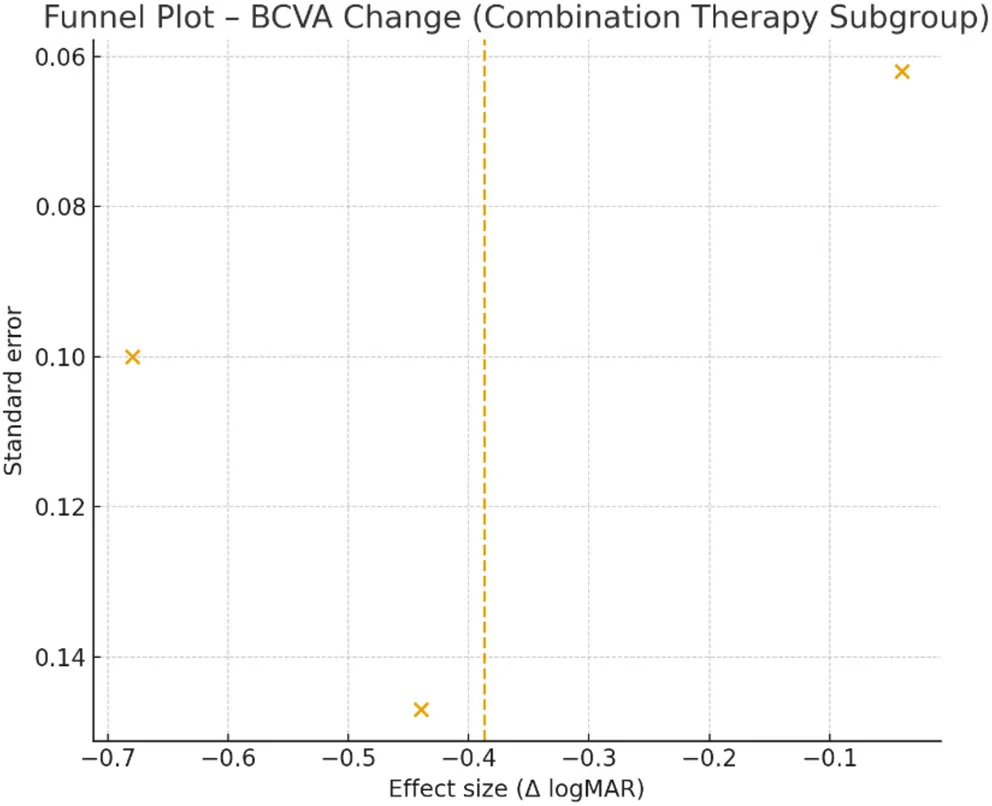
Funnel plot assessing publication bias in the meta‐analysis of BCVA change with combination therapy. Funnel plot illustrating the relationship between effect size (change in logMAR from baseline to final follow‐up) and standard error for studies included in the combination therapy subgroup. Each point represents an individual study. The vertical dashed line denotes the pooled effect estimate under the random‐effects model. The plot does not demonstrate marked asymmetry; however, due to the limited number of studies (*k* = 3), interpretation of publication bias is limited and findings should be viewed with caution.

## 4 DISCUSSION

### 4.1 Adult‐onset Coats disease as a distinct clinical entity

Adult‐onset Coats disease can be regarded as a clinically and biologically distinct phenotype within the Coats disease spectrum, rather than necessarily a delayed presentation of the paediatric phenotype. Across 35 studies encompassing 250 eyes, adults demonstrated a consistent demographic and clinical profile, with later onset, male predominance, predominantly unilateral involvement and a more localized presentation and frequently slower course at diagnosis as compared to paediatric patients (Hansraj et al., 2025; Park et al., 2016; Rishi et al., 2016; Shields & Shields, 2002; Zhou et al., 2024), with most eyes presenting at Shields Stage 2 or 3 and only a very small proportion at Stage 4, and no cases of Stage 5 disease identified in adults in the studies included in this review. While both paediatric and adult Coats disease share the hallmark features of retinal telangiectasia and intraretinal or subretinal lipid exudation, the adult form was consistently characterized by localized vascular abnormalities, a less aggressive angiogenic profile with lower rates of extensive exudation, neovascular complications and end‐stage retinal detachment, and lower rates of progression to total exudative retinal detachment or phthisis (Daruich et al., 2025; Shields & Shields, 2002).

Importantly, macular involvement, defined as the presence of macular exudation, cystoid macular oedema and/or macular telangiectasia identified on clinical examination or OCT, was present in 62% of adult cases, challenging the traditional belief that adult‐onset Coats disease is primarily a peripheral retinal disorder (Hansraj et al., 2025; Rishi et al., 2016; Xu et al., 2021; Zhou et al., 2024). Consistent with this, pooled OCT data from the included studies showed macular oedema or intraretinal cystoid spaces in approximately half of eyes, subretinal fluid in a similar proportion, and ERM and hyperreflective lipid exudates in a relevant minority, underscoring the central role of macular structural changes in visual morbidity. Regarding treatment, the majority of interventional series reported laser photocoagulation in over 80% of treated eyes, typically targeting peripheral telangiectatic vessels and areas of capillary nonperfusion on fluorescein angiography; some reports additionally described focal or sectoral laser to macular or juxtafoveal telangiectasia or lipid deposition when anatomically feasible, while avoiding direct foveal treatment. This macular predilection underscores the clinical relevance of early diagnostic imaging and potential intervention, as delayed treatment may compromise central visual outcomes. Collectively, these findings support the concept that adult‐onset Coats disease represents a distinct clinical phenotype with a chronic course and differential treatment response compared with paediatric disease, while leaving open the relative contributions of vascular, angiogenic and inflammatory mechanisms, which are further explored below.

### 4.2 Differential diagnosis and focal aneurysmal lesions in adult disease

Coats disease diagnosed in adulthood may overlap clinically and on imaging with other aneurysmal or telangiectatic retinal entities, particularly when lesions are central or when focal aneurysmal abnormalities predominate (Forte, Feo, et al., 2025). Perifoveal vascular anomalous complex (PVAC/PEVAC) and telangiectatic capillaries (TelCaps) represent important differentials in eyes with central aneurysmal lesions and exudation (Forte, Cattaneo, et al., 2025; Forte, Feo, et al., 2025); TelCaps can also occur secondary to broader retinal vascular disorders. Notably, Coats disease itself may be associated with focal aneurysmal lesions and telangiectatic capillaries, which may require targeted focal treatment in addition to broader ablation of leaking telangiectasia and nonperfused retina (Benlahbib et al., 2022; Luckie & Hamilton, 1994). Pragmatically, distinguishing diffuse telangiectatic disease with peripheral nonperfusion from focal aneurysmal lesions (often aided by multimodal imaging and, when available, indocyanine green angiography) can better inform treatment selection and expectations.

### 4.3 Comparison of adult‐onset and paediatric Coats disease: Clinical and therapeutic differences

This review points to a significant difference between adult and paediatric Coats disease in terms of onset, severity and therapeutic responsiveness. Paediatric disease typically presents before 10 years of age with profound subretinal exudation, more extensive and diffuse peripheral telangiectatic and aneurysmal vascular abnormalities that tend to progress if left untreated, rapid progression to advanced Shields Stage 3 (subtotal exudative retinal detachment) or Stage 4 (total retinal detachment, often with secondary complications such as neovascular glaucoma) and poor visual outcomes if not treated aggressively (Daruich et al., 2025; Shields & Shields, 2002). In contrast, patients with adult‐onset Coats disease more commonly present in Stage 2 (telangiectasia with intraretinal or subretinal exudation) or early Stage 3 (exudative retinal detachment that is often more localized), with exudation confined to the macula or peripheral retina and significantly lower incidence of total retinal detachment (Hansraj et al., 2025; Park et al., 2016; Rishi et al., 2016; Smithen et al., 2005). Notably, no cases of end‐stage (Stage 5) disease were reported in adults, in contrast to paediatric cohorts where Stage 5 disease has been reported in up to 25% of untreated cases (Daruich et al., 2025).

Therapeutically, paediatric Coats disease is largely driven by VEGF dysregulation, necessitating frequent anti‐VEGF injections and aggressive ablative therapies to mitigate neovascular complications (Heier & Boyer, 2018). In the adult population, laser photocoagulation alone achieved an anatomical success rate of 79.2%, indicating greater responsiveness to localized vascular ablation. Anti‐VEGF monotherapy was associated with high rates of anatomical response (88.9%), yet recurrences were frequent (66.7%), suggesting that VEGF blockade alone may be insufficient to achieve durable disease control in many adult eyes and that additional mechanisms, including chronic vascular instability and possibly inflammation, may contribute to disease maintenance. However, given selection bias, heterogeneous follow‐up and non‐standardized recurrence definitions, these comparative interpretations should be considered cautious.

It is conceivable that subclinical peripheral vascular abnormalities may have been present for years, or even congenitally, in some adults and only become clinically manifest or decompensate later in life; however, current evidence is insufficient to confirm this hypothesis. In contrast, in paediatric cases, VEGF overexpression has been postulated to be the central pathogenic factor driving exudation and neovascularization.

The substantial heterogeneity in functional (BCVA) outcomes observed across studies likely reflects differences in baseline macular involvement, disease stage at treatment initiation, treatment modality and follow‐up duration. Studies including eyes with chronic macular lipid exudation, cystoid macular oedema, fibrosis or ERM formation more frequently demonstrated limited visual recovery despite anatomical stabilization, whereas earlier‐stage disease without established macular structural damage was more often associated with visual stabilization. These factors should be considered when interpreting pooled functional outcomes in adult‐onset Coats disease, and underscore that stabilization rather than visual gain represents a realistic treatment goal in many cases.

### 4.4 Immunovascular mechanisms of exudation and macular oedema in adult‐diagnosed Coats disease

The heterogeneous treatment response suggests that multiple permeability pathways may contribute to exudation and macular oedema in Coats disease diagnosed in adulthood. While anti‐VEGF therapy can reduce leakage, responses may be incomplete or transient, and durable control frequently requires definitive treatment of telangiectatic vessels (laser or cryotherapy) and, in selected cases, adjunctive corticosteroids targeting downstream blood–retina barrier (BRB) dysfunction. Importantly, overt clinical inflammation is uncommon, and available aqueous cytokine studies are limited to small cohorts with restricted panels; therefore, inflammatory signalling should be considered a potential contributor to secondary oedema/exudation rather than a proven primary driver of the initiating vascular anomaly. However, the interpretation of intraocular cytokine levels should be approached with caution. Measurements may be influenced by prior intraocular procedures and sustained‐release corticosteroid treatments, and are further complicated by the very low physiological concentrations of many cytokines, which may affect assay reliability. Overall, while technically feasible, cytokine quantification in this context remains methodologically challenging.

There is emerging evidence that adult‐onset Coats disease may be at least partly driven by inflammation‐mediated endothelial dysfunction, rather than the VEGF‐dominant angiogenic mechanism classically described in paediatric cases (Krivosic et al., 2023; Wang et al., 2020). Aqueous humour analyses from adults with Coats disease have demonstrated marked elevations in IL‐6, tumour necrosis factor‐alpha (TNF‐α) and monocyte chemoattractant protein‐1 (MCP‐1), while VEGF levels were only mildly elevated or within normal physiological limits (Krivosic et al., 2023; Wang et al., 2020). These findings are hypothesis‐generating and require validation in larger, standardized cohorts.

This cytokine signature differs strikingly from paediatric Coats disease, in which VEGF concentrations are disproportionately high, driving aggressive exudation and neovascular complications (Daruich et al., 2025; Heier & Boyer, 2018). This biochemical divergence is further reflected clinically by the observation that corticosteroid therapy, particularly dexamethasone implant, appears beneficial in selected adult cases, either alone or as part of combination therapy with laser and anti‐VEGF agents.

Therefore, adult‐onset Coats disease may represent a chronic and possibly immune‐mediated yet predominantly localized, retinal microangiopathy, characterized by disruption of tight junctions—a mechanism that may overlap with, but is not specific to, VEGF‐driven disease—within the BRB, plasma lipid extravasation and telangiectatic vascular remodelling (Campochiaro et al., 2016). The strikingly unilateral presentation in most adult cases, together with the relative absence of overt inflammatory signs, argues against a systemic immune disorder and instead supports a focal retinal process in which inflammatory signalling may act as a contributory, modulatory factor rather than the sole pathogenic driver. This hypothesis is further supported by multimodal imaging: OCT consistently demonstrated hyperreflective foci that might correspond to lipid‐laden macrophages, and OCT‐A revealed deep capillary plexus flow deficits, both features that can be indicative of immune activation and endothelial barrier breakdown (Krivosic et al., 2023; Sakurada et al., 2018; Zhou et al., 2024). Collectively, current evidence points toward a multifactorial disease process in adult‐onset Coats disease, in which inflammation‐related mechanisms may contribute to disease activity alongside VEGF‐mediated vascular permeability. However, the available data remain limited, and the relative contribution of inflammatory versus angiogenic pathways cannot yet be clearly defined.

### 4.5 Role of multimodal imaging in redefining disease characteristics and the clinical spectrum

The advent of multimodal imaging has fundamentally transformed the understanding of adult‐onset Coats disease from a clinically inferred diagnosis to a quantifiable retinal vascular disorder with distinct angiographic and OCT signatures. Ultra‐widefield fluorescein angiography (UWFA) has proven particularly impactful, identifying extensive peripheral telangiectasia and capillary nonperfusion that may not be fully appreciated on fundus examination (Kumar, Chandra, et al., 2017; Park et al., 2016; Sakurada et al., 2018; Zhou et al., 2024). Across included studies, fluorescein angiography demonstrated telangiectasia in 75.7% of eyes and capillary dropout in 51.4%, reinforcing its diagnostic value.

OCT provided structural insights into macular involvement, revealing intraretinal cystoid changes in 51.4% and subretinal fluid in 51.4% of eyes. Notably, the presence of hyperreflective intraretinal deposits corresponding to lipid exudation may indicate persistent or subclinical vascular leakage that is not always apparent on ophthalmoscopic examination despite minimal clinical signs (Park et al., 2016; Xu et al., 2021; Zhou et al., 2024). Emerging evidence from OCT angiography (OCT‐A) further suggests that microaneurysms and capillary telangiectasia in adult‐onset Coats disease are associated with localized flow voids and abnormal deep capillary plexus morphology, consistent with endothelial barrier dysfunction rather than proliferative angiogenesis (Hoyek et al., 2025; Krivosic et al., 2023; Zhou et al., 2024).

As illustrated in Figure 5, even within the same patient examined on the same day, Coats disease may display markedly different anatomical features across imaging modalities and disease stages, which helps contextualize the heterogeneity of anatomical and functional outcomes reported across studies.

**FIGURE 5:**
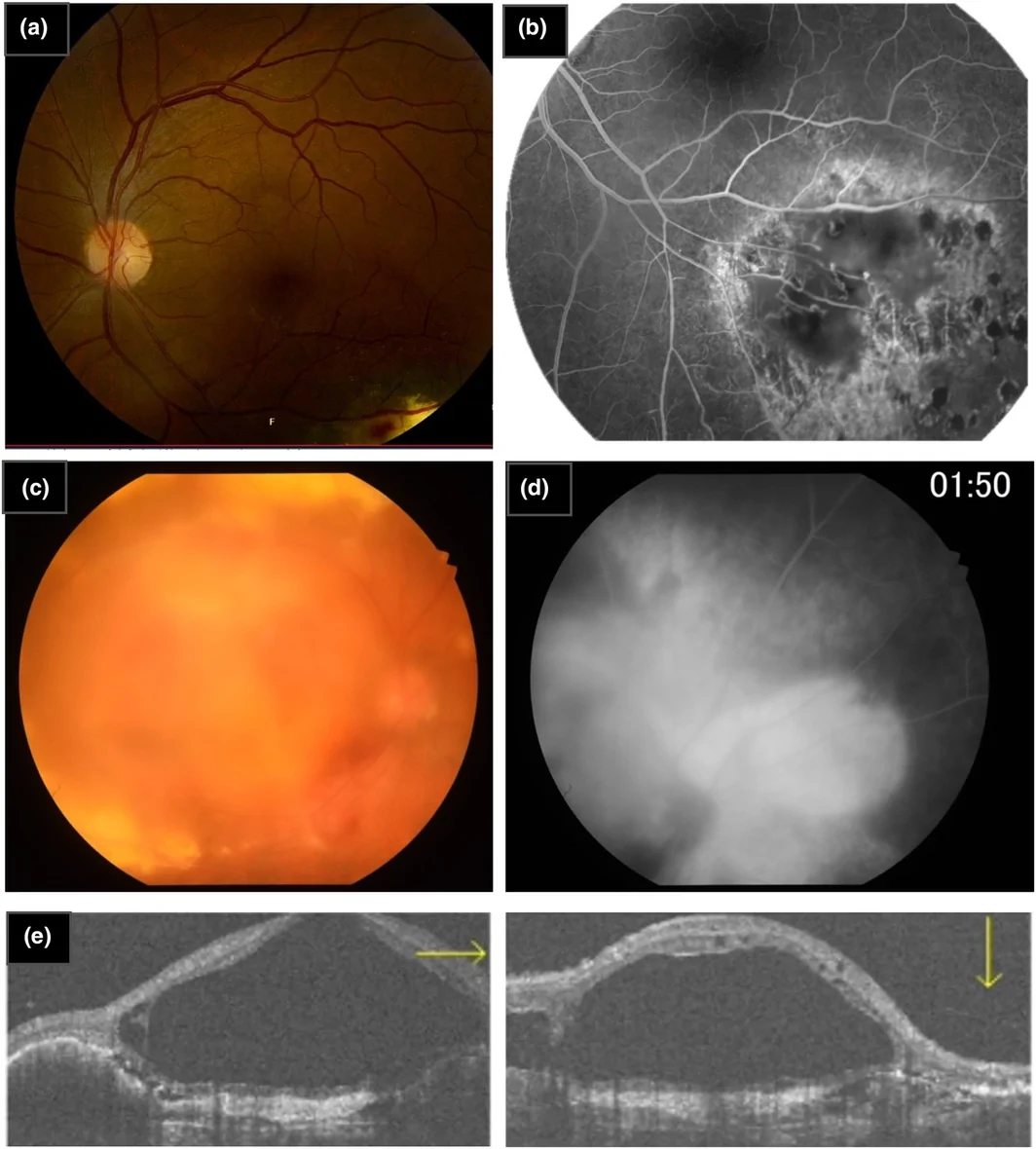
Multimodal retinal imaging in two cases of adult‐onset Coats disease. Case 1 (a–b) shows mild disease (Stage 2A) with focal extrafoveal changes sparing the fovea. Case 2 (c–e) illustrates advanced, long‐standing disease with extensive lipid exudation involving the posterior pole and peripheral retina (visualization partially limited by a subcapsular cataract). (a) Colour fundus photograph (Case 1) showing focal extrafoveal lipid exudation. (b) Fluorescein angiography (case 1; 1:50 min) demonstrating dye leakage from abnormal peripheral telangiectatic vessels. (c) Colour fundus photograph (Case 2) showing extensive lipid exudation at the posterior pole with macular detachment. (d) Fluorescein angiography (Case 2; 1:50 min) showing marked hyperfluorescence from diffuse leakage. (e) Spectral‐domain OCT (Case 2; two representative B‐scans) demonstrating bullous intraretinal fluid accumulation and disorganization of retinal layer architecture.

These imaging findings outperform traditional clinical staging systems by detecting subclinical disease activity, while enabling individualized treatment planning. They also support a shift toward a more mechanism‐informed imaging‐based assessment, incorporating vascular permeability, inflammatory biomarkers and capillary perfusion status (Shields & Shields, 2002). Rather than replacing the Shields classification, multimodal imaging should be viewed as complementary, as the Shields staging method remains useful for broad clinical stratification but may not fully capture disease activity, lesion distribution, or the potential for relapse in adult‐onset disease. We also incorporated recent Acta Ophthalmologica evidence on foveal/subfoveal vascular pathologies and prognostic imaging biomarkers (Brockmann et al., 2026; Yuksel et al., 2025), supporting the relevance of complementary foveal descriptors alongside traditional staging in both paediatric and adult‐diagnosed disease.

Thus, advanced imaging is not merely diagnostic but provides additional structural and functional information that may have prognostic relevance and help guide personalized treatment strategies in adult‐onset Coats disease.

### 4.6 Therapeutic implications: Targeting vascular instability, inflammation, and leakage

The therapeutic landscape of adult‐onset Coats disease is undergoing re‐evaluation in light of evolving mechanistic insights. Nevertheless, laser photocoagulation of telangiectatic vessels remains the cornerstone of treatment, based on the available evidence, as it effectively induces vascular closure and reduces exudation. (Park et al., 2016; Rishi et al., 2016). Laser monotherapy achieved anatomical success (i.e. near‐complete resolution of exudation and retinal reattachment without need for additional interventions) in 79.2% of treated eyes, particularly in early Shields Stage 2 disease (Park et al., 2016). However, in eyes with extensive exudation or subretinal fluid, laser monotherapy alone was often insufficient, likely reflecting persistent leakage from inadequately treated or newly manifest telangiectatic vessels, and possibly in part additional mechanisms such as low‐grade inflammatory‐mediated vascular permeability, rather than inflammation alone (Chen et al., 2020; Hansraj et al., 2025).

Anti‐VEGF therapy, while highly effective in paediatric Coats disease, demonstrated mixed results in adults. Although bevacizumab and other anti‐VEGF reduced macular oedema, its effects were frequently transient, and recurrence rates were notably higher compared to laser‐treated eyes (66.7% vs. 10.4%). This temporal pattern may partly reflect the pharmacodynamic limitations of anti‐VEGF therapy, which requires repeated administration to maintain VEGF suppression, rather than definitively indicating that VEGF is not involved in disease activity in adults. Accordingly, VEGF‐related vascular permeability may coexist with other contributory mechanisms in adult‐onset disease, rather than acting as the sole pathological driver (Hansraj et al., 2025; Park et al., 2016).

Intravitreal corticosteroids emerged as a particularly effective modality in studies that evaluated their use, with dexamethasone implants demonstrating robust anatomical responses even in refractory cases (Chen et al., 2020). Corticosteroids act by reducing cytokine‐mediated endothelial permeability and stabilizing the BRB, directly addressing the proposed inflammatory pathophysiology. Combination therapy—integrating laser ablation with corticosteroid and/or anti‐VEGF injections—produced the most consistent anatomical resolution, reflecting the multifactorial nature of disease mechanisms in adults. In selected cases, a stepwise or even triple‐therapy approach may therefore be required, combining definitive treatment of peripheral telangiectasia with adjunctive measures to suppress ongoing leakage from residual or secondary abnormal vessels contributing to macular oedema.

Taken together, the available evidence supports a pragmatic, combination‐based approach in many adult‐diagnosed cases, with definitive ablation of leaking telangiectasia and areas of nonperfusion by laser or cryotherapy, complemented by adjunctive pharmacotherapy tailored to macular oedema severity and the imaging and biomarker profile. However, given selection bias, small sample sizes and heterogeneous endpoints, the current literature does not establish the superiority of any single drug class, and prospective comparative studies are needed.

### 4.7 Prognosis and the emergence of a chronic, relapsing vascular phenotype

Adult‐onset Coats disease demonstrates a fundamentally different prognostic trajectory from its paediatric counterpart. In paediatric cases, late‐stage presentation and rapid progression more commonly leads to irreversible and severe complications such as retinal detachment, neovascular glaucoma and eventually phthisis (Daruich et al., 2025; Shields & Shields, 2002). Conversely, patients with adult‐onset Coats disease show a slower disease course, with better structural preservation of the retina and lower rates of progression to advanced stages, also because the earlier‐stage disease presentation allows for earlier therapeutic intervention.

Overall, based on the available evidence assessed in this review, anatomical success was achieved in 52.2% of all treated eyes, and more than half maintained stability or improvement in visual acuity. However, recurrence of disease activity occurred in 19.3% of eyes, primarily within the first year after treatment, indicating that adult‐onset Coats disease may be a chronic relapsing vasculopathy rather than a one‐time exudative event in some cases. It should be noted that recurrence has been variably defined across studies and may include reappearance or progression of telangiectatic vascular lesions, renewed or increased lipid exudation, recurrent macular oedema or subretinal fluid; therefore, reported recurrence rates should be interpreted cautiously. In addition, incomplete initial ablation or differences in follow‐up intensity may contribute to recurrence being detected and reported.

This underscores the importance of active monitoring of adult‐onset Coats disease patients. Importantly, recurrence was not limited to advanced‐stage disease; even eyes presenting with minimal exudation exhibited reactivation after initial treatment, supporting the concept of persistent localized vascular instability and ongoing disease susceptibility rather than inevitable progression to advanced pathology.

### 4.8 Proposed conceptual model of adult‐onset Coats disease pathogenesis

We propose a conceptual framework to illustrate potential mechanisms involved in adult‐onset Coats disease, rather than a definitive pathogenetic model. In this framework, adult‐diagnosed Coats disease is viewed as a chronic retinal telangiectatic vasculopathy characterized by endothelial dysfunction and microvascular instability, with downstream BRB dysfunction leading to exudation/macular oedema through VEGF‐related permeability and potentially contributory inflammatory signalling. Unlike paediatric Coats disease, which is dominated by VEGF‐induced angiogenesis and rapid exudation, the adult form may involve a more complex interplay of vascular and non‐vascular factors, potentially including inflammatory signalling, although current biomarker evidence in adults remains limited and hypothesis‐generating (Park et al., 2016; Rishi et al., 2016).

### 4.9 Limitations of current evidence

While this systematic review highlights novel mechanistic and clinical insights into adult‐onset Coats disease, several limitations inherent to the available literature must be acknowledged. First, most included studies were retrospective case reports or small observational case series, which are highly susceptible to selection bias and lack standardized outcome reporting (Page & Moher, 2021; Park et al., 2016; Rishi et al., 2016; Shukla et al., 2008). The predominance of single‐centre studies further limits external validity and introduces potential institutional treatment bias.

Second, there was substantial heterogeneity in diagnostic criteria, imaging modalities and treatment protocols across studies, precluding uniform comparison of therapeutic efficacy and functional outcomes (Chen et al., 2020; DerSimonian & Laird, 2015; Hansraj et al., 2025; Rishi et al., 2016; Zhou et al., 2024). Only a minority of studies reported paired visual acuity in standardized logMAR format, necessitating conversion from Snellen or qualitative vision measures, which may introduce imprecision despite use of validated methods (Kumar, Chandra, et al., 2017; Takeyama et al., 2012).

Third, no randomized controlled trials were identified, and nearly all interventional studies utilized multimodal treatment regimens, making it difficult to isolate the effect of individual therapeutic agents such as anti‐VEGF drugs or corticosteroids (Chen et al., 2020; Heier & Boyer, 2018). Follow‐up durations also varied widely, with several studies reporting only short‐term outcomes, which may underestimate recurrence rates and long‐term retinal remodelling (Campochiaro et al., 2016; Hansraj et al., 2025).

Fourth, assessment of inflammatory biomarkers was limited to a small subset of studies, restricting the ability to draw firm conclusions regarding the role of immunoinflammatory mechanisms in disease pathogenesis (Krivosic et al., 2023; Wang et al., 2020). Although aqueous humour cytokine analysis supports an inflammatory profile in some adults with Coats disease, these findings are based on limited sample sizes and should be interpreted as hypothesis‐generating rather than confirmatory, requiring validation in larger, prospective cohorts with standardized molecular endpoints.

Fifth, pooled anatomical outcome estimates should be interpreted cautiously given heterogeneity in outcome definitions, baseline stage, macular involvement and treatment selection across studies.

Finally, publication bias is likely, as studies with positive therapeutic outcomes are more frequently reported, while negative or inconclusive results may remain unpublished (Page & Moher, 2021). Although funnel plot analysis did not demonstrate marked asymmetry, interpretative confidence is limited by the small number of studies eligible for quantitative synthesis.

### 4.10 Future directions and translational opportunities

Future research could prioritize the validation of the inflammatory paradigm through prospective, biomarker‐driven studies that quantify aqueous and vitreous cytokine levels and correlate them with disease activity and treatment response (Krivosic et al., 2023; Wang et al., 2020). These molecular insights may further enable precision medicine approaches, allowing more individualized therapy based on inflammatory and angiogenic disease profiles.

Advances in multimodal imaging, particularly OCT‐A and ultra‐widefield angiography, present an opportunity to develop mechanism‐based staging systems that better reflect disease activity than traditional anatomical classifications (Spaide et al., 2018; Zhou et al., 2024). Imaging biomarkers such as deep capillary plexus flow deficits and BRB permeability changes may facilitate earlier intervention and guide treatment escalation.

Emerging therapies—including intravitreal corticosteroids, IL‐6 inhibitors and lipid‐modulating agents—hold promise for targeting the possible underlying immunovascular mechanisms of adult‐onset Coats disease. In parallel, artificial intelligence–based image analysis may revolutionize disease monitoring and prognostication, enabling personalized treatment intervals and automated detection of subclinical reactivation (Daich Varela et al., 2023).

## 5 CONCLUSION

This systematic review provides an overview of adult‐onset Coats disease, highlighting a phenotype diagnosed in adulthood within the Coats disease spectrum rather than assuming a late manifestation of the paediatric form (Campochiaro, 2015; Shields & Shields, 2002). There is evidence suggesting that its pathophysiology involves endothelial dysfunction with downstream BRB breakdown, with inflammatory signalling potentially contributing alongside VEGF‐mediated permeability. This mechanistic profile may explain the more protracted course in adults and the frequent use of multimodal approaches combining vascular ablation with adjunctive pharmacotherapy, including corticosteroids in selected cases, rather than reliance on anti‐VEGF monotherapy alone (DerSimonian & Laird, 1986; Park et al., 2016; Rishi et al., 2016).

Multimodal imaging has been central in redefining disease characterization, enabling earlier detection, stratification of risk and targeted treatment planning. Collectively, the data may support a treatment paradigm shift from stage‐based management to mechanism‐based personalized therapy (Campochiaro, 2015; Sacconi et al., 2019). Recognizing adult‐onset Coats disease require long‐term monitoring and biomarker‐driven treatment strategies (Ferris et al., 1982; Hansraj et al., 2025; Rishi et al., 2016).

Future research may focus on assessing inflammatory pathways as potential therapeutic targets, and prospective, controlled studies can optimize treatment algorithms.

## FUNDING INFORMATION

This research received no specific grant from any funding agency in the public, commercial or not‐for‐profit sectors.

## Data Availability

No new datasets were generated. All data supporting the findings of this study are included in the manuscript and its tables.

## References

[aos70149-bib-0001] Abatli, S. & Shweiki, S.Z. (2025) Coats' disease in adulthood with preserved vision after intravitreal aflibercept injection combined with laser photocoagulation: a case report. BMC Ophthalmology, 25, 8. 10.1186/s12886-024-03829-4 PMC11706013 39762784

[aos70149-bib-0002] Alsaggaf, K. , Jalloun, M. , Alkhotani, W. & Albeedh, M. (2020) Three‐year results of management of adult‐onset Coats' disease by possibly targeting placental growth factor. Cureus, 12, e10652. 10.7759/cureus.10652 PMC7586352 33133822

[aos70149-bib-0003] Amoroso, F. , Miere, A. , Colantuono, D. , Pedinielli, A. , Cohen, S.Y. & Souied, E. (2024) Navigated focal laser of macular telangiectasia type I (adult‐onset Coats' disease). Ophthalmic Surgery, Lasers & Imaging Retina, 55, 545–551. 10.3928/23258160-20240412-02 38917396

[aos70149-bib-0004] Amro, M. , Haydar, A.A. , El‐Khoury, S. , Tomey, K.F. , Cherfan, G. & Khoueir, Z. (2018) Acute angle‐closure glaucoma as a first presentation of Coats' disease: a case report. Journal of Glaucoma, 27, e33–e36. 10.1097/IJG.0000000000000838 29176335

[aos70149-bib-0005] Benlahbib, M. , Semoun, O. , Amoroso, F. , Colantuono, D. & Souied, E.H. (2022) Multimodal imaging of unusual macular macroaneurysm rupture after navigated retinal laser in a patient with adult onset Coats disease. Am J Ophthalmol Case Rep, 26, 101458. 10.1016/j.ajoc.2022.101458 PMC8914334 35282602

[aos70149-bib-0006] Black, G.C. , Perveen, R. , Bonshek, R. , Cahill, M. , Clayton‐Smith, J. , Lloyd, I.C. et al. (1999) Coats' disease of the retina (unilateral retinal telangiectasis) caused by somatic mutation in the NDP gene: a role for norrin in retinal angiogenesis. Human Molecular Genetics, 8, 2031–2035. 10.1093/hmg/8.11.2031 10484772

[aos70149-bib-0007] Booth, A. (2013) PROSPERO at one year: an evaluation of its utility. Systematic Reviews, 2, 4. 10.1186/2046-4053-2-4 PMC3563608 23320413

[aos70149-bib-0008] Brockmann, C. , Muller, B. , Schonfeld, S. , Winterhalter, S. , Zeitz, O. & Joussen, A.M. (2026) Long‐term outcome of Coats' disease: implications for the classification of foveal vascular pathologies. Acta Ophthalmologica, 104, e104–e111. 10.1111/aos.17554 PMC12803690 40616365

[aos70149-bib-0009] Campochiaro, P.A. (2015) Molecular pathogenesis of retinal and choroidal vascular diseases. Progress in Retinal and Eye Research, 49, 67–81. 10.1016/j.preteyeres.2015.06.002 PMC4651818 26113211

[aos70149-bib-0010] Campochiaro, P.A. , Aiello, L.P. & Rosenfeld, P.J. (2016) Anti‐vascular endothelial growth factor agents in the treatment of retinal disease: from bench to bedside. Ophthalmology, 123, S78–S88. 10.1016/j.ophtha.2016.04.056 27664289

[aos70149-bib-0011] Chen, Q.S. , Liang, S.Y. , Wang, X.Z. , Hu, C.L. , She, J.T. & Li, Z. (2020) Efficacy and safety outcomes of intravitreal dexamethasone implant therapy for the treatment of adult Coats' disease. Journal of Ophthalmology, 9131908. 10.1155/2020/9131908 PMC7547358 33062317

[aos70149-bib-0012] Cheung, C.M.G. & Arnold, J.J. (2012) Idiopathic macular telangiectasia type 1 (adult‐onset Coats disease). Survey of Ophthalmology, 57, 387–407.

[aos70149-bib-0013] Coats, G. (1908) Forms of retinal disease with massive exudation. Royal London Ophthalmic Hospital Reports, 17, 440–525.

[aos70149-bib-0014] Daich Varela, M. , Sen, S. , De Guimaraes, T.A.C. , Kabiri, N. , Pontikos, N. , Balaskas, K. et al. (2023) Artificial intelligence in retinal disease: clinical application, challenges, and future directions. Graefe's Archive for Clinical and Experimental Ophthalmology, 261, 3283–3297. 10.1007/s00417-023-06052-x PMC10169139 37160501

[aos70149-bib-0015] Daruich, A. , Bremond‐Gignac, D. , Aziz, A. , Couret, C. , Gallice, M. , Krivosic, V. et al. (2025) National protocol for diagnosis and treatment of Coats disease: summary for attending and primary care physicians. Journal Français d'Ophtalmologie, 48. 10.1016/j.jfo.2025.104539 40339459

[aos70149-bib-0016] Dave, A.D. , Thavikulwat, A.T. , De Silva, T. , Wiley, H.E. , Keenan, T.D.L. , Wong, W.T. et al. (2022) Longitudinal characterization and treatment response of retinal arterial macroaneurysms in adult‐onset coats disease. Am J Ophthalmol Case Rep, 27, 101647. 10.1016/j.ajoc.2022.101647 PMC9289817 35859699

[aos70149-bib-0017] DerSimonian, R. & Laird, N. (1986) Meta‐analysis in clinical trials. Controlled Clinical Trials, 7, 177–188. 10.1016/0197-2456(86)90046-2 3802833

[aos70149-bib-0018] DerSimonian, R. & Laird, N. (2015) Meta‐analysis in clinical trials revisited. Contemporary Clinical Trials, 45, 139–145. 10.1016/j.cct.2015.09.002 PMC4639420 26343745

[aos70149-bib-0019] Elnahry, A.G. , Sallam, E.M. , Guirguis, K.J. , Talbet, J.H. & Abdel‐Kader, A.A. (2019) Vitrectomy for a secondary epiretinal membrane following treatment of adult‐onset Coats' disease. Am J Ophthalmol Case Rep, 15, 100508. 10.1016/j.ajoc.2019.100508 PMC6624194 31334386

[aos70149-bib-0020] Falagas, M.E. & Pitsouni, E.I. (2008) Comparison of PubMed, Scopus, Web of Science, and Google Scholar. FASEB Journal, 22, 338–342. 10.1096/fj.07-9492LSF 17884971

[aos70149-bib-0021] Fatihoglu, O.U. , Ayhan, Z. , Ozturk, T. , Kavukcu, S. , Yaman, A. & Saatci, A.O. (2025) Place of dexamethasone implant as an adjunctive treatment in Coats' disease: a retrospective single‐center study. BMC Ophthalmology, 25, 435. 10.1186/s12886-025-04279-2 PMC12309173 40731271

[aos70149-bib-0022] Feng, J. & Zhao, T. (2017) 'Differences in intraocular cytokine profiles between pediatric and adult Coats disease. Investigative Ophthalmology & Visual Science, 58, 3099–3105.

[aos70149-bib-0023] Fernandes, B.F. & Odashiro, A.N. (2011) Coats‐like retinal vascular changes in systemic diseases. Survey of Ophthalmology, 56, 196–203.

[aos70149-bib-0024] Ferris, F.L., 3rd , Kassoff, A. , Bresnick, G.H. & Bailey, I. (1982) New visual acuity charts for clinical research. American Journal of Ophthalmology, 94, 91–96. 7091289

[aos70149-bib-0025] Forte, P. , Cattaneo, J. , Fontana, V. , Dupas, B. , Forte, G. , Castro‐Farias, D. et al. (2025) Telangiectatic capillaries in retinal vein occlusion: incidence, topography, and risk factors. Ophthalmol Sci, 5, 100754. 10.1016/j.xops.2025.100754 PMC11985091 40212937

[aos70149-bib-0026] Forte, P. , Feo, A. , Sarraf, D. , Romano, M.R. & Nicolo, M. (2025) Current insights and challenges in the management of perifoveal vascular anomalous complex (PVAC): a systematic review of multimodal imaging features and treatment strategies. Eye (London, England), 39, 449–459. 10.1038/s41433-024-03544-w PMC11794436 39702788

[aos70149-bib-0027] Forte, P. , Paques, M. , Cattaneo, J. , Dupas, B. , Castro‐Farias, D. , Girmens, J.F. et al. (2025) Perifoveal vascular anomalous complex and telangiectatic capillaries: an overview of two entities potentially sharing a common pathophysiology. Survey of Ophthalmology, 70, 369–379. 10.1016/j.survophthal.2025.01.007 39824296

[aos70149-bib-0028] Garoon, R.B. & Shields, C.L. (2020) Adult‐onset Coats disease: clinical features and treatment outcomes. Current Opinion in Ophthalmology, 31, 167–174.

[aos70149-bib-0029] Gawecki, M. (2021) Idiopathic peripheral retinal telangiectasia in adults: a case series and literature review. Journal of Clinical Medicine, 10, 1767. 10.3390/jcm10081767 PMC8072906 33921646

[aos70149-bib-0030] Goel, N. & Kumar, V. (2018) Adult‐onset Coats' disease: clinical spectrum and treatment outcomes. International Ophthalmology, 38, 1665–1671.

[aos70149-bib-0031] Goel, N. , Kumar, V. , Seth, A. , Raina, U.K. & Ghosh, B. (2011) Role of intravitreal bevacizumab in adult onset Coats' disease. International Ophthalmology, 31, 183–190. 10.1007/s10792-011-9436-x 21437759

[aos70149-bib-0032] Greenhalgh, T. (2014) Integrative literature reviews and meta‐analyses. BMJ (Clinical Research ed.), 349, g7094.

[aos70149-bib-0033] Hansraj, S. , Raval, V. , Jalali, S. , Sahoo, N. & Das, A.V. (2025) Clinical presentation and treatment outcomes of adult‐onset Coats disease. Journal of Vitreoretinal Diseases, 9, 43–49. 10.1177/24741264241286580 PMC11556389 39539824

[aos70149-bib-0034] He, Y.G. & Wang, H. (2016) Elevated inflammatory cytokines in adult Coats disease: implications for pathogenesis. Molecular Vision, 22, 457–465.

[aos70149-bib-0035] Heier, J.S. & Boyer, D.S. (2018) VEGF as a driver of retinal exudation in pediatric Coats disease. Retina (Philadelphia, Pa.), 38, 2059–2066.

[aos70149-bib-0036] Higgins, J.P.T. (2003) Measuring inconsistency in meta‐analyses. BMJ (Clinical Research ed.), 327, 557–560. 10.1136/bmj.327.7414.557 PMC192859 12958120

[aos70149-bib-0037] Higgins, J.P.T. & Thomas, J. (2019) Cochrane handbook for systematic reviews of interventions. Chichester, UK: Cochrane.

[aos70149-bib-0038] Higgins, J.P.T. & Thompson, S.G. (2002) Quantifying heterogeneity in a meta‐analysis. Statistics in Medicine, 21, 1539–1558. 10.1002/sim.1186 12111919

[aos70149-bib-0039] Hoyek, S. , Patel, N.A. , Parikh, A.A. , Yaghy, A. , Mukai, S. & Baumal, C.R. (2025) Comparison of adult and pediatric eyes with Coats disease using multimodal imaging. Journal of Vitreoretinal Diseases, 9, 659–666. 10.1177/24741264251355635 PMC12277311 40698348

[aos70149-bib-0040] Kim, J.H. & Kim, J.W. (2016) Clinical characteristics of adult‐onset Coats disease and comparison with pediatric patients. Eye (London), 30, 438–444.

[aos70149-bib-0041] Koh, Y.T. & Sanjay, S. (2013) Successful outcome of adult‐onset Coats' disease following retinal laser photocoagulation. Oman J Ophthalmol, 6, 206–207. 10.4103/0974-620X.122280 PMC3872574 24379559

[aos70149-bib-0042] Krivosic, V. , Mecê, P. , Dulière, C. , Lavia, C. , Zegrari, S. , Tadayoni, R. et al. (2023) Abnormalities in the retinal capillary plexuses in coats disease in adulthood on optical coherence tomography angiography. Retina (Philadelphia, Pa.), 43, 1514–1524. 10.1097/IAE.0000000000003834 37199396

[aos70149-bib-0043] Kumar, A. , Kumar, P. , Ambiya, V. & Mishra, S.K. (2022) Subthreshold micropulse laser for adult onset Coats' associated exudative maculopathy. European Journal of Ophthalmology, 32, NP29–NP31. 10.1177/11206721211005691 33765847

[aos70149-bib-0044] Kumar, K. , Raj, P. , Chandnani, N. & Agarwal, A. (2019) Intravitreal dexamethasone implant with retinal photocoagulation for adult‐onset Coats' disease. International Ophthalmology, 39, 465–470. 10.1007/s10792-018-0827-0 29380183

[aos70149-bib-0045] Kumar, P. & Kumar, V. (2017) Vitrectomy for epiretinal membrane in adult‐onset Coats' disease. Indian Journal of Ophthalmology, 65, 1046–1048. 10.4103/ijo.IJO_322_17 PMC5678313 29044085

[aos70149-bib-0046] Kumar, V. , Chandra, P. & Kumar, A. (2017) Ultra‐wide field imaging in the diagnosis and management of adult‐onset Coats' disease. Clinical & Experimental Optometry, 100, 79–82. 10.1111/cxo.12418 27476647

[aos70149-bib-0047] Kumar, V. , Kumar, P. , Garg, G. & Damodaran, S. (2017) Vitrectomy for full‐thickness macular hole in adult‐onset Coats' disease. Indian Journal of Ophthalmology, 65, 1246–1248. 10.4103/ijo.IJO_546_17 PMC5700610 29133668

[aos70149-bib-0048] Lange, C. , Feltgen, N. , Junker, B. , Schulze‐Bonsel, K. & Bach, M. (2009) Resolving the clinical acuity categories “hand motion” and “counting fingers” using the Freiburg Visual Acuity Test (FrACT). Graefe's Archive for Clinical and Experimental Ophthalmology, 247, 137–142. 10.1007/s00417-008-0926-0 18766368

[aos70149-bib-0049] Ledesma‐Gil, G. , Moreno Andrade, A.B. & Shields, C.L. (2022) Secondary vasoproliferative tumor in adult‐onset Coats disease. Canadian Journal of Ophthalmology, 57, 69–70. 10.1016/j.jcjo.2021.03.002 33798454

[aos70149-bib-0050] Li, S. & Li, Y. (2021) Distinct clinical course of adult‐onset Coats disease. British Journal of Ophthalmology, 105, 509–514.

[aos70149-bib-0051] Luckie, A.P. & Hamilton, A.M. (1994) Adult Coats' disease in branch retinal vein occlusion. Australian and New Zealand Journal of Ophthalmology, 22, 203–206. 10.1111/j.1442-9071.1994.tb01717.x 7818879

[aos70149-bib-0052] Methley, A.M. & Campbell, S. (2014) PICO, PICOS and SPIDER: a comparison study. BMC Health Services Research, 14, 50. 10.1186/s12913-014-0579-0 PMC4310146 25413154

[aos70149-bib-0053] Mino, A. , Mitamura, Y. , Katome, T. , Semba, K. , Egawa, M. & Naito, T. (2015) Case of adult‐onset Coats' disease with epiretinal membrane treated with 25‐gauge pars plana vitrectomy. Journal of Medical Investigation, 62, 85–88. 10.2152/jmi.62.85 25817290

[aos70149-bib-0054] Munn, Z. & Peters, M.D.J. (2018) Systematic review or scoping review? Guidance for authors. BMC Medical Research Methodology, 18, 143. 10.1186/s12874-018-0611-x PMC6245623 30453902

[aos70149-bib-0055] Mustafi, D. & Stacey, A.W. (2022) Unilateral peripheral vascular retinal disease associated with a developmental arcade vessel anomaly in adult‐onset Coats' disease. Am J Ophthalmol Case Rep, 25, 101408. 10.1016/j.ajoc.2022.101408 PMC8851077 35198825

[aos70149-bib-0056] Otani, T. , Yasuda, K. , Aizawa, N. , Sakai, F. , Nakazawa, T. & Shimura, M. (2011) Over 10 years follow‐up of Coats' disease in adulthood. Clinical Ophthalmology, 5, 1729–1732. 10.2147/OPTH.S27938 PMC3245190 22205832

[aos70149-bib-0057] Ouzzani, M. & Hammady, H. (2016) Rayyan—a web and mobile app for systematic reviews. Systematic Reviews, 5, 210. 10.1186/s13643-016-0384-4 PMC5139140 27919275

[aos70149-bib-0058] Page, M.J. & Moher, D. (2021) PRISMA 2020 explanation and elaboration. BMJ (Clinical Research ed.), 372, n160. 10.1136/bmj.n160 PMC8005925 33781993

[aos70149-bib-0059] Park, J.J. , Gole, G.A. , Finnigan, S. & Vandeleur, K. (1999) Late presentation of a unilateral sporadic retinoblastoma in a 16‐year‐old girl. Australian and New Zealand Journal of Ophthalmology, 27, 365–368. 10.1046/j.1440-1606.1999.00217.x 10571403

[aos70149-bib-0060] Park, S. , Cho, H.J. , Lee, D.W. , Kim, C.G. & Kim, J.W. (2016) Intravitreal bevacizumab injections combined with laser photocoagulation for adult‐onset Coats' disease. Graefe's Archive for Clinical and Experimental Ophthalmology, 254, 1511–1517. 10.1007/s00417-015-3233-6 26631136

[aos70149-bib-0061] Rishi, E. , Rishi, P. , Appukuttan, B. , Uparkar, M. , Sharma, T. & Gopal, L. (2016) Coats' disease of adult‐onset in 48 eyes. Indian Journal of Ophthalmology, 64, 518–523. 10.4103/0301-4738.190141 PMC5026078 27609165

[aos70149-bib-0062] Sacconi, R. , Giuffre, C. , Corbelli, E. , Borrelli, E. , Querques, G. & Bandello, F. (2019) Emerging therapies in the management of macular edema: a review. F1000Research, 8, 1413. 10.12688/f1000research.19198.1 PMC6694451 31448093

[aos70149-bib-0063] Sakurada, Y. , Freund, K.B. & Yannuzzi, L.A. (2018) Multimodal imaging in adult‐onset Coats' disease. Ophthalmology, 125, 485. 10.1016/j.ophtha.2017.12.007 29566865

[aos70149-bib-0064] Sen, M. , Shields, C.L. , Honavar, S.G. & Shields, J.A. (2019) Coats disease: an overview of classification, management and outcomes. Indian Journal of Ophthalmology, 67, 763–771. 10.4103/ijo.IJO_841_19 PMC6552590 31124484

[aos70149-bib-0065] Shields, C.L. & Kaliki, S. (2013) Retinal capillary hemangioma: clinical spectrum and outcomes in 57 patients. Ophthalmology, 120, 1832–1839.

[aos70149-bib-0066] Shields, J.A. & Shields, C.L. (2002) Review: coats disease: the 2001 LuEsther T. Mertz lecture. Retina, 22, 80–91. 10.1097/00006982-200202000-00014 11884883

[aos70149-bib-0067] Shienbaum, G. & Tasman, W.S. (2006) Coats disease: a lifetime disease. Retina, 26, 422–424. 10.1097/01.iae.0000238545.44132.a2 16603961

[aos70149-bib-0068] Shukla, D. , Chakraborty, S. , Behera, U.C. & Kim, R. (2008) Vitrectomy for epimacular membrane secondary to adult‐onset Coats' disease. Ophthalmic Surgery, Lasers & Imaging, 39, 239–241. 10.3928/15428877-20080501-15 18556951

[aos70149-bib-0069] Smithen, L.M. & Brown, G.C. (2005) Coats' disease diagnosed in adulthood. Ophthalmology, 112, 1072–1078. 10.1016/j.ophtha.2004.12.038 15882905

[aos70149-bib-0070] Smithen, L.M. , Brown, G.C. , Brucker, A.J. , Yannuzzi, L.A. , Klais, C.M. & Spaide, R.F. (2005) Coats' disease diagnosed in adulthood. Ophthalmology, 112, 1072–1078. 10.1016/j.ophtha.2004.12.038 15882905

[aos70149-bib-0071] Spaide, R.F. , Fujimoto, J.G. , Waheed, N.K. , Sadda, S.R. & Staurenghi, G. (2018) Optical coherence tomography angiography. Progress in Retinal and Eye Research, 64, 1–55. 10.1016/j.preteyeres.2017.11.003 PMC6404988 29229445

[aos70149-bib-0072] Synthesis, J. B. I. Manual for Evidence . (2020) Joanna Briggs Institute Critical Appraisal Tools.

[aos70149-bib-0073] Takeyama, M. , Iwaki, M. & Zako, M. (2012) Intravitreal injection of bevacizumab to treat a macular edema caused by Leber's miliary aneurysm. Case Rep Ophthalmol, 3, 392–395. 10.1159/000345455 PMC3531946 23275795

[aos70149-bib-0074] Vazquez‐Pulido, N. , Laliena Santamaria, J.L. , Del Buey Sayas, M.A. , Burdeus Gomez, R. & Osan Tello, M. (2007) [Adult‐onset of recurrent Coats disease]. Archivos de la Sociedad Española de Oftalmología, 82, 555–558. 10.4321/s0365-66912007000900008 17846946

[aos70149-bib-0075] Wang, K.Y. & Cheng, C.K. (2011) A combination of intravitreal bevacizumab injection with tunable argon yellow laser photocoagulation as a treatment for adult‐onset Coats' disease. Journal of Ocular Pharmacology and Therapeutics, 27, 525–530. 10.1089/jop.2011.0088 21951171

[aos70149-bib-0076] Wang, Y. , Fan, H. , Gao, K. , He, W. & Tao, Y. (2020) Levels of cytokines in the aqueous humor guided treatment of refractory macular edema in adult‐onset coats' disease. BMC Ophthalmology, 20, 261. 10.1186/s12886-020-01474-1 PMC7329434 32605612

[aos70149-bib-0077] Wells, G.A. (2011) The Newcastle–Ottawa Scale (NOS) for assessing quality in nonrandomized studies.

[aos70149-bib-0078] Xu, X. , Essilfie, J. , Gong, Y. , Yu, S.Q. & Freund, K.B. (2021) Resolution of foveal lipid deposition in adult‐onset Coats disease with combined focal laser photocoagulation and anti‐VEGF therapy. Ophthalmic Surgery, Lasers & Imaging Retina, 52, 396–399. 10.3928/23258160-20210628-07 34309430

[aos70149-bib-0079] Yuksel, M. , Ozdek, S. , Zeydanli, E.O. , Atalay, H.T. , Ozdemir, H.B. , Hasanreisoglu, C.M. et al. (2025) Development of subfoveal nodule in Coats' disease: prevalence, stages, prognosis, and treatment. Acta Ophthalmologica. 10.1111/aos.70055 41354905

[aos70149-bib-0080] Zhang, L.L. , Ke, Y.F. , Wang, W. , Shi, X.Y. , Hei, K.W. & Li, X.R. (2018) The efficacy of conbercept or ranibizumab intravitreal injection combined with laser therapy for Coats' disease. Graefes Archive for Clinical and Experimental Ophthalmology, 256, 1339–1346. 10.1007/s00417-018-3949-1 PMC6006222 29549425

[aos70149-bib-0081] Zheng, X.X. & Jiang, Y.R. (2014) The effect of intravitreal bevacizumab injection as the initial treatment for Coats' disease. Graefes Archive for Clinical and Experimental Ophthalmology, 252, 35–42. 10.1007/s00417-013-2409-1 23873253

[aos70149-bib-0082] Zhou, W. , Zhou, H. , Liu, Y.Y. , Li, M.X. , Wu, X.H. , Liang, J. et al. (2024) Multimodal imaging diagnosis and analysis of prognostic factors in patients with adult‐onset Coats disease. International Journal of Ophthalmology, 17, 1469–1476. 10.18240/ijo.2024.08.12 PMC11286439 39156792

[aos70149-bib-0083] Zhou, Y. & Sun, M. (2024) Adult Coats disease: clinical features and visual outcomes. Retina, 44, 345–352.

